# The Effect of the Ratio of Gamma Aminobutyric Acid-Producing *Saccharomyces cerevisiae* DL6–20 and *Kluyveromyces marxianus* B13–5 Addition on Cheese Quality

**DOI:** 10.3389/fmicb.2022.900394

**Published:** 2022-06-23

**Authors:** Shan Li, Yan Zhang, Xu Li, Pingping Yin, Tengbin Wang, Yandie Li, Kaili Zhang, Huayang Sheng, Shiling Lu, Hua Ji, Zhexin Fan, Baokun Li

**Affiliations:** ^1^School of Food Science and Technology, Key Laboratory of Xinjiang Phytomedicine Resource and Utilization of Ministry of Education, Shihezi University, Shihezi, China; ^2^Henan Shuanghui Investment & Development Co., Ltd., Luohe, China; ^3^Zhoukou Vocational College of Arts and Science, Zhoukou, China; ^4^Guangdong Yikewei Biotech Co., Ltd., Guangzhou, China; ^5^Xinjiang Uygur Autonomous Region Analysis and Testing Research Institute, Xinjiang, China

**Keywords:** yeast, GABA, cheese, physical and chemical indicators, flavor compounds, multivariate statistical analysis

## Abstract

Kazakh cheese is a traditional dairy product in Xinjiang, China. The function and potential probiotic characteristics of *Saccharomyces cerevisiae* DL6–20 and *Kluyveromyces marxianus* B13–5 in Kazakh cheese and its contribution to cheese fermentation was studied. In this study, the effect of the addition ratio of gamma aminobutyric acid (GABA)-producing *S. cerevisiae* DL6–20 and *K. marxianus* B13–5 on cheese quality was investigated. Cheeses were prepared by fermentations with a total of six treatments: comercial culture alone as control (CS), a combination with one yeast, either; *K. marxianus* B13–5 (CSM); *S. cerevisiae* DL6–20 (CSS); and three different proportions of this two yeasts (CSM:CSS 1:1, 1:2, 2:1). We measured the GABA content of cheese, as well as basic physical and chemical indicators, microbial content, free amino acid (FAA) content, texture, and flavor compound content. The total FAA content of mixed bacteria fermentation was higher than that of the single bacteria alone. The GABA content CSM:CSS 1:2 GABA content was 0.114 g/100 g, CSM:CSS 2:1 GABA content was 0.12 g/100 g, CSM:CSS1:1 content of GABA produced in the late ripening period of cheese was the highest, reaching 0.189 g/100 g and the number of LAB and yeasts in CSM:CSS 1:1 was higher than that of other cheeses. The mixed-strain fermentation generally produced cheeses with a higher protein content than that of the single-strain fermentation in the late stage of the maturation process, especially the protein content of CSM:CSS 1:1 during the ripening period, when the protein content was highest at day 50. CSM:CSS 1:1 had a low moisture content, making it easy to store. With the exception of water and protein content, there is no significant difference in other physical and chemical indicators. CSM:CSS 1:1 contributed to the formation of cheese texture. In addition, multivariate statistical analysis indicated that mixed-strain fermentation was beneficial to the production of cheese aroma, with the aroma production performance of CSM:CSS 1:2 and CSM:CSS 2:1 found to be better than that of CSM: CSS 1:1.

## Highlights

- The effect of mixed-strain fermentation on cheese quality.
- Mixed fermentation showed higher protein and total FAA contents than single bacterial fermentation.
- Mixed fermentation was beneficial to the production of cheese aromas than single bacterial fermentation.
- Mixed fermentations with strains *K. marxianus* B13–5 and *S. cerevisiae* DL6–20 (1:1) showed higher GABA and low moisture contents.
- Mixed fermentations with strains *K. marxianus* B13–5 and *S. cerevisiae* DL6–20 (1:1) is conducive to storage and industrial production.

## Introduction

GABA is a non-protein amino acid that functions as the main inhibitory neurotransmitter in the central nervous system of mammals. GABA has been shown to improve brain function, and prevent or reduce anxiety, depression, insomnia, and memory loss. It also stimulates the immune system, prevents inflammation, reduces high blood pressure and ameliorates diabetes, and regulates energy metabolism (Dhakal et al., 2012; Karimian et al., 2020). Studies in mice have shown that GABA at dose levels of 100 and 150 μg improved improve discrimination learning ability (Ishikawa and Saito, 1978). Exposure to a high altitude environment and ingestion of 100 mg GABA in subjects with acrophobia has been shown to increase alpha brain waves and decrease immunoglobulin A levels, thereby promoting relaxation and reducing anxiety (Abdou et al., 2006). Daily intake of fermented milk containing 10 mg of GABA for 12 weeks can also help control blood pressure levels in patients with mild hypertension (Inoue et al., 2003).

GABA occurs naturally in many plant foods, and is present at high levels in fermented products, especially fermented dairy products (Diana et al., 2014; Park et al., 2021). Since lactic acid bacteria (LAB) are recognized as safe microorganisms, most studies have focused on their GABA production capacity (Perpetuini et al., 2020). However, few studies have focused on yeast, even though these strains play an important role in the production wine, bread, dairy products and many other fermented foods (Hittinger et al., 2018). Yeast have ability to metabolize proteins, lipids and organic acids, allowing it to grow on the surface of cheese and promote the formation of cheese flavor and texture (Perkins et al., 2020). Sechi reported that the GABA production of *Saccharomyces cerevisiae* JBCC-A74 was 0.33 g/L (Sechi et al., 2014), whereas Perpetuini et al. (2020) demonstrated that the GABA production of *Kluyveromyces marxianus* K326 was 7.78 ± 1.88 mg/L.

Xinjiang is a vast and sparsely populated region in China, with a large area of pastureland, and unique natural conditions for the development of the dairy industry. The Kazak people mainly live in the northern part of Xinjiang. Most of people live a nomadic life, and their main diet is meat and dairy products. Kazak cheese is made using the fermentation of cow’s milk and has many features including having good nutritional components and flavor, and ease of transport and storage. It is a must-have for Kazakh herdsmen when out with grazing animals and entertaining guests (Xu et al., 2020). The raw material of Xinjiang Kazak cheese is produced within the Tianshan Range. After the milk is heated and boiled, it is then spontaneous fermented to produce acid and curd. The whey would be filtered with gauze, and the fresh cheese is placed on a bamboo board for 30–90 days of spontaneous ripening, at an average temperature between 5 and 10°C with a humidity of 85–90%.To study the effects of GABA-producing yeast on cheese, GABA-producing yeast strains *S. cerevisiae* DL6–20 and *K. marxianus* B13–5, from Xinjiang dairy products were used to make experimental cheese, with cheese made using a commercial starter being used as the control group. We aimed to study the effect of different proportions of GABA-producing yeast on cheese quality, in order to obtain the best proportion and provide a basis for industrial production of functional cheese.

## Materials and Methods

### Sources of Gamma Aminobutyric Acid-Producing Yeast

A commercial starter YO-MIX187 (*Streptococcus salivarius* ssp. *Thermophilus* and *Lactobacillus delbrueckii* ssp. bulgaricus; Danisco A/S) was used as a source of LAB in this study. The yeast strains *K. marxianus* B13–5 and *S. cerevisiae* DL6–20 (NCBI strain accession numbers MW287159 and MW287161, respectively), which produce high levels of GABA and good cheese flavor, were used in this study (Li et al., 2021.

### Cheese Preparation and Sampling

Six 4L batches of standardized milk (Garden Dairy Co., Ltd., Xinjiang, China) were pasteurized at 65°C for 30 min (Li et al., 2020). The cheeses of the control groups were made by using 2.5% of commercial starter. Five separate cheese batches were also prepared using the 2.5% (v/v) commercial fermentation starter until the pH dropped to 5.0, followed by fermentation with two yeast strains added to the mixture in the following proportions: *K. marxianus* B13–5 alone, *S. cerevisiae* DL6–20 alone, *K. marxianus* B13–5: *S. cerevisiae* DL6–20 1:1 (CSM: CSS 1:1), *K. marxianus* B13–5: *S. cerevisiae* DL6–20 1:2 (CSM: CSS 1:2), and *K. marxianus* B13–5: *S. cerevisiae* DL6–20 2:1 (CSM: CSS 2:1).

The commercial starter was added to the milk at a concentration of approximately 1.0 × 10^7^ cfu/mL and activated by heating to 43°C. Yeast strains were activated in yeast extract peptone dextrose medium (YPD) medium at 28°C and the viable count reached approximately 1.0 × 10^7^ cfu/mL for further processing. Overnight cultures were harvested (3,000 × *g*, 15 min, 4°C), washed twice with phosphate buffer solution (PBS) solution (pH = 7), and resuspended in milk. Curd formation was performed under sealed conditions. At the end of the fermentation process, the curd block was cut into 1-cm cubes and strained using four layers of gauze to separate the whey before being placed in a compression mold. After the whey was removed, 1% salt was added to the curd, which was then dried at room temperature. After the cheeses is collected, put it into a vacuum bag for storage, and the storage temperature is 10°C.

Samples were obtained after 0, 10, 20, 30, 40 and 50 days. The sample was then placed in a 50 mL centrifuge tube, frozen in liquid nitrogen, and stored at −80°C until to analysis.

### Microbiological Analysis

Colony formation assays were used to determine the microorganism content of cheese samples obtained 0, 10, 20, 30, 40, and 50 days after ripening. Samples (10 g) of grated cheese were placed in 90 mL of sterile 0.85% (w/v) saline for 5 min before serial dilutions with 9 mL sterile normal saline (Kocak et al., 2020). To determine the number of LAB colonies, diluted samples were incubated in MRS medium (Qingdao New Hope BioTechnology Co., Ltd., Qingdao, China) at 37°C for 36 h. To count yeast colonies, diluted samples were cultured in YPD medium containing chloramphenicol (0.01%, w/v) at 28°C for 48 h. In both cases, the colonies were counted on a plate containing 30–300 colonies and the results expressed as the log of the colony forming units per gram of cheese (log cfu/g).

### Texture Evaluation

A texture analyzer (TA.Xtplus, Micro Stable System Co., Tianjin, China) was used for analysis of the following texture characteristics: hardness, springing, cohesiveness, chewiness, gumminess and gumminess resilience. A typical two-port compression test was performed with the P-5 probe. Before testing, the cheese samples were brought to room temperature (Xia et al., 2020). The test conditions are as follows: texture profile analysis (TPA); pre-test speed (5 mm/s), test speed (2 mm/s), post-test speed (2.0 mm/s), strain 45%.

### Determination of Basic Physical and Chemical Indicators

The lactic acid content was measured titration according to the method described by Li et al. (2020). Briefly, the pH value of the cheese was measured using a calibrated electronic digital pH meter (Thermo Fisher Scientific, Rambo Co., Ltd., Shanghai, China). The moisture content of the cheese sample was measured overnight in a constant-temperature drying oven at 105°C by atmospheric drying method; the protein content was measured using the Kjeldahl method (KjeIMaster K-375; BÜCHI Laboratechnik AG, Switzerland; McCarthy et al., 2017). All tests were repeated three times.

### Determination of Gamma Aminobutyric Acid Content and Free Amino Acid Content

The cheese samples were processed according to the method described by Chen et al. (2016), and the GABA content was determined by high performance liquid chromatography (HPLC) according to the method described by Kantachote et al. (2017). Cheese samples (1 g) were placed in 3 mL ultrapure water, vortexed, and incubated in a water bath at 40°C for 1 h before centrifugation for 20 min at 6,000 × *g*/min. A sample (1 mL) of the supernatant was then freeze-dried. Subsequently, 1 mL ethanol-water-triethylamine (4:4:2) solution and 80 μL ethanol-water-triethylamine-phenylisothiocyanate (6:1:1:1) were added to the residue. The mixture was incubated for 20 min in the dark at room temperature and filtered through a 0.22 μm membrane. The content of GABA in the fermentation broth was determined by HPLC under the following conditions: Shimazu C18 chromatographic column (4.6 mm × 250 mm; Dima Technology Co., Ltd., Guangzhou, China). Samples (injection volume 10 μL) were eluted using mobile phase A [1.4 mm sodium acetate buffer containing 0.1% triethylamine and 6% acetonitrile (pH 6.1)] and mobile phase B (60% acetonitrile) under the following gradient elution program: (0–100%) mobile phase B for 50 min; 0% mobile phase B for 10 min. The flow rate was 1 mL/min and the detection wavelength was 254 nm.

The FAA content of samples was determined using an amino acid analyzer according to the method described by Buňka et al. (2009). Amino acids were detected at 440 nm (proline and hydroxyproline) and 570 nm (all other FAAs), and the amino acids were quantified by comparing the peak area of the standard mixture with that obtained using a solution of known concentration.

### Assay of Volatile Compounds

The volatile compounds in cheese samples were extracted and analyzed by solid phase microextraction-gas chromatography-mass spectrometry (SPME/GC-MS; Palo Alto, CA, U.S. Agilent Co., Ltd., Palo Alto, CA, United States) according to the method described by Zheng et al. (2018). Each cheese sample (1.5 g) was mixed with 0.2 g anhydrous sodium sulfate and 0.2 g sodium chloride plus an internal standard (1 μL 0.856 mg/mL 2-methyl-3-heptanone). The sample was then placed in a vial with a 20 mL headspace and immediately covered with a polytetrafluoroethylene (PTFE) membrane. The sample was equilibrated in a 45°C water bath for 20 min before a 1 cm stable flexible 50/30 μm SPME fiber (DVB/CAR/PDMS 50/30 μm; Supelco, Bellefonte, PA, United States) was inserted (Zheng et al., 2018; Wang et al., 2020). Volatile compounds were absorbed at 45°C for 30 min prior to chromatographic analysis using an Innowax-Wax column (30 m × 0.25 mm, 0.25 μm; Agilent Technologies) under the following conditions: manual injection; inlet temperature 230°C; heating program, 40°C for 4 min, followed by increased temperature to 100°C at a rate of 5°C/min and held for 2 min, followed by increased temperature to 180°C at a rate of 6°C/min, and finally, increased temperature to 230°C at a rate of 8°C/min and held for 2 min. Helium was used as the carrier gas at a constant flow rate of 0.8 mL/min, and sample without splitting. The mass spectrometry conditions were as follows: mass detector operating in 70 eV electron impact mode; ion source temperature 200°C; interface temperature 250°C; detector temperature 280°C; full scan mode; mass range 20–350 amu; 5 scans/s; and no solvent delay. Flavor compounds were identified by comparing the retention indexes with those in the National Institute of Standards and Technology mass spectrum library. The aroma compounds with a matching score >80 (maximum 100) were determined using the area normalization method in which the concentration of each compound was equal to the ratio of the peak area of the compound to the peak area of the internal standard.

### Statistical Analysis

All data were expressed as the means ± standard deviation (SD) of three trials for each sample. Significant differences between the groups were assessed with Duncan multiple range tests and least significant difference tests using IBM SPSS statistics software version 22 (IBM Corp., Armonk, NY, United States) *P* < 0.05 was considered to indicate statistical significance. TBtools was used to generate a heatmap for analysis of the changes in flavor characteristics during the fermentation of the cheese samples. Principal component analysis (PCA), orthogonal partial least squares discrimination analysis (OPLS-DA), hierarchical cluster analysis (HCA) and variable importance of projection (VIP) values were used to reduce data dimensions using SIMCA 14.1 software (Biometric Software Developer Umetrics, Umea, Sweden).

## Results and Discussion

### Microorganism Content

**FIGURE 1:**
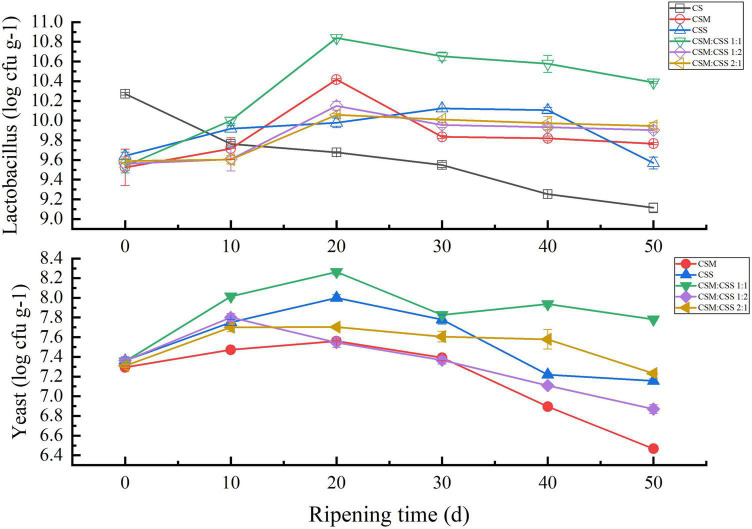
Comparative analysis of the microorganism content of the cheeses. CS, CSM, CSS, CSM:CSS 1:1, CSM:CSS 1:2 and CSM:CSS 2:1 represent cheeses fermented using the commercial starter (Control group), *K. marxianus* B13–5, *S. cerevisiae* DL6–20, *K. marxianus* B13–5: *S. cerevisiae* DL6–20 1:1, *K. marxianus* B13–5: *S. cerevisiae* DL6–20 1:2, and *K. marxianus* B13–5: *S. cerevisiae* DL6–20 2:1. Error bars indicate standard deviations.

During the cheese ripening process, the numbers of viable yeast and LAB in the experimental group first increased and then decreased, while no decreased was detected in the control group cheese (Figure 1). The number of viable LAB in the cheese in the experimental group was higher than that in the control group, mainly because yeast has a positive effect on the survival of LAB (Ferreira and Viljoen, 2003). Yeast produces metabolites and growth factors (vitamins and amino acids), thereby promoting bacterial growth (Larpin-Laborde et al., 2011; Cogan Timothy et al., 2014). As shown in Figure 1, among the five experimental groups, the numbers of viable LAB and yeast in CSM:CSS 1:1 were higher than those in the other four groups, indicating that the two yeast strains promote growth most effectively at this inoculation ratio. In addition, yeast produces large amounts of amino acids during cheese ripening (Fröhlich-Wyder et al., 2019). The highest total FAA content of CSM:CSS 1:1 in Figure 1 can illustrate this result.

### Cheese Texture

Texture is one of the main quality attributes of cheese, and its development is inextricably linked with the biochemical and physical changes that occur during the maturation process (Lucey et al., 2005). To evaluate the influence of fermentation strains on cheese texture, it was evaluated characteristics such as hardness, springiness, cohesiveness, gumminess, chewiness and chewiness resilience (Table 1). The hardness of the six cheeses was inversely proportional to the moisture content. CSM:CSS 1:1 has the lowest moisture content and the hardest texture. This mixture is suitable for making hard cheeses and has a longer shelf life. CMS had the highest protein content and noticeable high hardness. This is mainly due to the fact that the hardness itself is related to the protein content of cheese, with increased protein content associated with harder cheese (Adda et al., 1982). Cohesiveness indicates the degree to which the cheese sticks together in the mouth (Diezhandino et al., 2016). An increase in the interaction between protein and water leads to the deterioration of the cohesiveness of the cheese (Moreira et al., 2018). The cohesiveness of the cheese in the control group tended to decrease in the late stage of maturation, mainly due to a decrease in the interaction of the protein matrix with water contained in the cheese. The chewiness of the cheese showed a similar trend to the hardness, which is consistent with the results reported by Zheng et al. (2012). The springiness in the six groups decreased slightly during the maturation process, whereas the chewiness resilience increased slightly. Lobato-Calleros et al. (2008) reported that fresh cheese proteins are highly cross-linked in a three-dimensional network and show high resistance to deformation, which means that cheese is more elastic. The springiness of the six cheeses was not significantly different (*P* > 0.05). The chewiness resilience of CS and CSM:CSS 1:1 cheeses was significantly higher than that of other cheeses (*P* < 0.05), but the hardness of CS was significantly lower than that of CSM:CSS 1:1. The results indicate that the cheese in the CSM:CSS 1:1 group has a denser structure than that in the CS group, although it is similar to that of the cheeses in the CSM, CSS, CSM:CSS 1:2, and CSM:CSS 2:1 groups, but has higher resistance to deformation.

**TABLE 1:** Comparative analysis of the textures of the cheeses.

	Day (d)	Hardness (N)	Springiness	Cohesiveness	Gumminess (N)	Chewiness (N)	Chewiness resilience
CS	0	6.494 ± 0.592^eA^	0.951 ± 0.037^aA^	0.396 ± 0.027^abA^	3.543 ± 0.173^cA^	3.317 ± 0.45^dBC^	0.077 ± 0.003^bA^
	10	7.656 ± 0.672^dD^	0.934 ± 0.054^aAB^	0.443 ± 0.046^aA^	4.707 ± 0.201^bBC^	3.86 ± 0.4562^cdB^	0.089 ± 0.004^aA^
	20	9.577 ± 0.561^cB^	0.933 ± 0.042^aA^	0.365 ± 0.017^bA^	4.992 ± 0.372^abCD^	4.323 ± 0.673^bcCD^	0.088 ± 0.002^aA^
	30	11.752 ± 0.743^bA^	0.906 ± 0.056^abA^	0.406 ± 0.029^abAB^	5.255 ± 0.295^abC^	4.824 ± 0.489^abC^	0.071 ± 0.003^cBC^
	40	12.334 ± 0.378^bC^	0.837 ± 0.047^bcA^	0.438 ± 0.031^aAB^	5.382 ± 0.401^aD^	5.623 ± 0.527^aBC^	0.082 ± 0.001^bAB^
	50	14.022 ± 0.462^aB^	0.800 ± 0.035^cA^	0.448 ± 0.042^aA^	5.581 ± 0.374^aA^	5.718 ± 0.437^aA^	0.093 ± 0.004^aA^
CSM	0	6.955 ± 0.317^eA^	0.982 ± 0.046^aA^	0.375 ± 0.039^bcA^	2.229 ± 0.189^cA^	2.194 ± 0.27^cC^	0.067 ± 0.004^cA^
	10	9.033 ± 0.247^dBC^	0.977 ± 0.029^abA^	0.396 ± 0.036^abA^	3.911 ± 0.057^bC^	3.821 ± 0.148^bB^	0.067 ± 0.002^cB^
	20	10.896 ± 0.339^cAB^	0.919 ± 0.027^abA^	0.433 ± 0.018^aA^	4.326 ± 0.512^bD^	3.966 ± 0.351^bD^	0.087 ± 0.014^abA^
	30	12.834 ± 0.539^bA^	0.87 ± 0.059^abA^	0.428 ± 0.008^aAB^	5.49 ± 0.308^aBC^	4.779 ± 0.463^abC^	0.093 ± 0.003^aA^
	40	15.528 ± 0.857^aA^	0.865 ± 0.112^bA^	0.337 ± 0.02^cB^	5.233 ± 0.509^aD^	4.566 ± 0.994^abC^	0.062 ± 0.004^cC^
	50	16.201 ± 0.355^aA^	0.895 ± 0.033^abA^	0.364 ± 0.023^bcA^	5.879 ± 0.515^aA^	5.273 ± 0.444^aA^	0.075 ± 0.011^bcBC^
CSS	0	6.346 ± 0.205^dA^	1.550 ± 0.101^aA^	0.534 ± 0.161^aA^	3.973 ± 0.397^cA^	3.213 ± 0.206^dBC^	0.049 ± 0.005^bB^
	10	8.175 ± 0.583^cCD^	0.982 ± 0.017^aA^	0.399 ± 0.051^abA^	4.480 ± 0.787^bcBC^	4.399 ± 0.789^cB^	0.064 ± 0.007^aB^
	20	11.712 ± 0.601^bAB^	0.951 ± 0.003^aA^	0.393 ± 0.012^abA^	5.782 ± 0.336^abcBC^	5.497 ± 0.329^bcBCD^	0.057 ± 0.003^abC^
	30	12.130 ± 0.306^bA^	0.95 ± 0.01^aA^	0.398 ± 0.017^abAB^	6.335 ± 0.334^abBC^	6.031 ± 0.33^abBC^	0.059 ± 0.005^abC^
	40	14.805 ± 0.412^aAB^	0.949 ± 0.107^aA^	0.397 ± 0.076^abAB^	7.052 ± 0.631^aBC^	6.776 ± 0.945^aABC^	0.065 ± 0.004^aC^
	50	15.280 ± 1.041^aAB^	0.899 ± 0.084^aA^	0.37 ± 0.044^bA^	7.139 ± 0.557^aA^	6.393 ± 0.892^abA^	0.066 ± 0.008^aC^
CSM:CSS 1:1	0	6.905 ± 0.822^eA^	1.937 ± 0.103^aA^	0.394 ± 0.023^aA^	3.906 ± 0.504^bA^	7.92 ± 0.585^aA^	0.08 ± 0.007^abA^
	10	10.867 ± 0.814^dA^	0.852 ± 0.033^bB^	0.419 ± 0.021^aA^	7.491 ± 0.69^abA^	6.395 ± 0.825^aA^	0.094 ± 0.011^aA^
	20	11.427 ± 0.623^dAB^	0.958 ± 0.016^bA^	0.431 ± 0.016^aA^	7.933 ± 0.431^abA^	7.597 ± 0.333^aA^	0.079 ± 0.005^bAB^
	30	12.847 ± 0.617^cA^	0.938 ± 0.042^bA^	0.429 ± 0.048^aAB^	8.091 ± 0.905^abA^	7.915 ± 0.808^aA^	0.08 ± 0.007^abB^
	40	14.352 ± 0.733^bAB^	0.919 ± 0.021^bA^	0.388 ± 0.086^aAB^	9.293 ± 0.314^aA^	8.627 ± 0.28^aA^	0.093 ± 0.009^abA^
	50	16.559 ± 0.32^aA^	0.896 ± 0.143^bA^	0.372 ± 0.047^aA^	5.829 ± 0.598^abA^	5.272 ± 0.813^aA^	0.085 ± 0.001^abAB^
CSM:CSS 1:2	0	6.644 ± 0.489^dA^	1.019 ± 0.145^aA^	0.428 ± 0.043^aA^	3.693 ± 0.349^dA^	3.795 ± 0.891^eB^	0.069 ± 0.011^abA^
	10	11.003 ± 0.212^cA^	0.93 ± 0.01^aAB^	0.434 ± 0.036^aA^	5.650 ± 0.45^cB^	5.258 ± 0.448^dAB^	0.073 ± 0.002^aB^
	20	11.588 ± 1.044^bcAB^	0.975 ± 0.061^aA^	0.408 ± 0.05^aA^	6.760 ± 0.525^bAB^	6.222 ± 0.738^cdAB^	0.067 ± 0.005^abBC^
	30	12.164 ± 0.303^bA^	0.919 ± 0.055^aA^	0.372 ± 0.032^aB^	6.932 ± 0.425^bAB^	6.756 ± 0.56^bcAB^	0.067 ± 0.002^abBC^
	40	13.413 ± 0.217^aBC^	0.978 ± 0.023^aA^	0.416 ± 0.016^aAB^	8.496 ± 0.405^aAB^	7.805 ± 0.877^abAB^	0.06 ± 0.001^bC^
	50	14.102 ± 0.841^aB^	0.907 ± 0.034^aA^	0.372 ± 0.037^aA^	8.590 ± 0.662^aA^	8.606 ± 0.958^aA^	0.075 ± 0.002^aBC^
CSM:CSS 2:1	0	6.113 ± 0.381^dA^	1.037 ± 0.127^aA^	0.396 ± 0.07^aA^	2.795 ± 0.338^dA^	2.926 ± 0.719^cC^	0.064 ± 0.006^bAB^
	10	10.143 ± 0.279^cAB^	0.959 ± 0.055^aA^	0.437 ± 0.03^aA^	4.869 ± 0.348^cBC^	4.663 ± 0.268^bB^	0.07 ± 0.004^abB^
	20	12.367 ± 1.16^bA^	0.98 ± 0.021^aA^	0.436 ± 0.027^aA^	5.846 ± 0.863^bcBC^	5.732 ± 0.905^abBC^	0.068 ± 0.002^abBC^
	30	12.512 ± 0.482^bA^	1.062 ± 0.177^aA^	0.478 ± 0.06^aA^	6.441 ± 0.692^bBC^	5.785 ± 0.046^abBC^	0.075 ± 0.007^aB^
	40	14.815 ± 0.498^aAB^	0.923 ± 0.064^aA^	0.487 ± 0.023^aA^	7.696 ± 0.349^aBC^	6.359 ± 1.148^aBC^	0.078 ± 0.006^aB^
	50	15.788 ± 0.891^aAB^	0.88 ± 0.06^aA^	0.483 ± 0.089^aA^	7.708 ± 0.833^aA^	7.115 ± 0.746^aA^	0.064 ± 0.005^bC^

*CS, CSM, CSS, CSM:CSS 1:1, CSM:CSS 1:2 and CSM:CSS 2:1 represent cheeses fermented by Commercial Stater (Control group), K. marxianus B13–5, S. cerevisiae DL6–20, K. marxianus B13–5: S. cerevisiae DL6–20 1:1, K. marxianus B13–5: S. cerevisiae DL6–20 1: 2, K. marxianus B13–5: S. cerevisiae DL6–20 2:1. Data are expressed as the mean ± standard deviation from three replicate analyses (n = 3) of three replicate samples. A–D indicates that there are significant differences between the samples from different strains obtained on the same day (P < 0.05), a–e indicates that there are significant differences between the samples from different days obtained on the same strains (P < 0.05).*

### Basic Physical and Chemical Indicators

**FIGURE 2:**
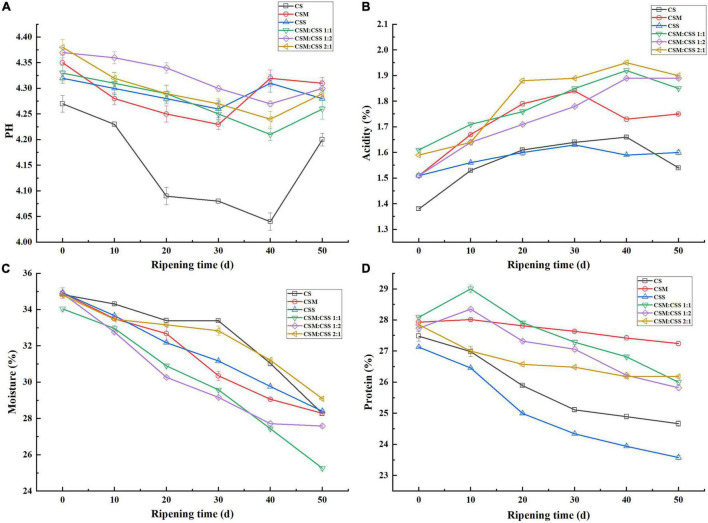
Comparative analysis of the physical and chemical indicators of the cheeses. CS, CSM, CSS, CSM:CSS 1:1, CSM:CSS 1:2 and CSM:CSS 2:1 represent cheeses fermented using the commercial starter (Control group), *K. marxianus* B13–5, *S. cerevisiae* DL6–20, *K. marxianus* B13–5: *S. cerevisiae* DL6–20 1:1, *K. marxianus* B13–5: *S. cerevisiae* DL6–20 1: 2, *K. marxianus* B13–5: *S. cerevisiae* DL6–20 2:1. Error bars indicate standard deviations. **(A–D)** The changes in pH, acidity, moisture, and protein content during storage, respectively.

The physical and chemical indicators of ripening cheese (moisture, pH, lactic acid content and protein content) are shown in Figure 2. During the ripening process, the pH of the six cheeses generally decreased and then increased. The decrease in pH during the early stage of cheese ripening was due to the metabolism of lactose to lactic acid through glycolysis, whereas the subsequent increase in pH was due to the metabolism of lactic acid and the microbiota that hydrolyze the protein to form alkaline products (Xia et al., 2020). This increase in pH was caused by nitrogen compounds, which were the key to the ripening of these cheeses. The pH values in the experimental groups (CSM, CSS, CSS:CSM 1:1, CSS:CSM 1:2, CSS:CSM 2:1) were significantly higher (*P* < 0.05) than that of the control group (CS), which may be because yeast metabolizes lactic acid and produces H_2_0 And CO_2_, which reduces acid compounds that increase pH (Cholet et al., 2007). In the single-strain fermented cheese ripened for 30–50 days, the pH first increased and then decreased, while the pH of the mixed-strain fermented cheeses continued to decrease until 40 days. This may be due to the co-existence of yeast strains providing more factors to promote the growth of LAB, and the pH value still shows a downward trend at 40 days (Adesulu-Dahunsi et al., 2020). The study found that the change in lactic acid content was opposite to the change in pH value during fermentation, which is consistent with the results of Öründü and Tarakçı (2021).

The moisture content of cheese not only affects the yield of cheese, but also plays a vital role in the quality of cheese. As the cheese ripens, the moisture content decreases, usually due to the evaporation of free water (Pappa et al., 2006), which can also be confirmed by the water droplets observed in a vacuum bag. After 50 days of cheese ripening, CSM:CSS 2:1, CS, CSS and CMS had a relatively high moisture content, possibly due to the production of water during the proteolysis mediated by the different bacteria (Lawrence et al., 1987). CSM:CSS 1:1 has the lowest moisture content, which is convenient for storage and transportation, and is more suitable for commercial production.

**FIGURE 3:**
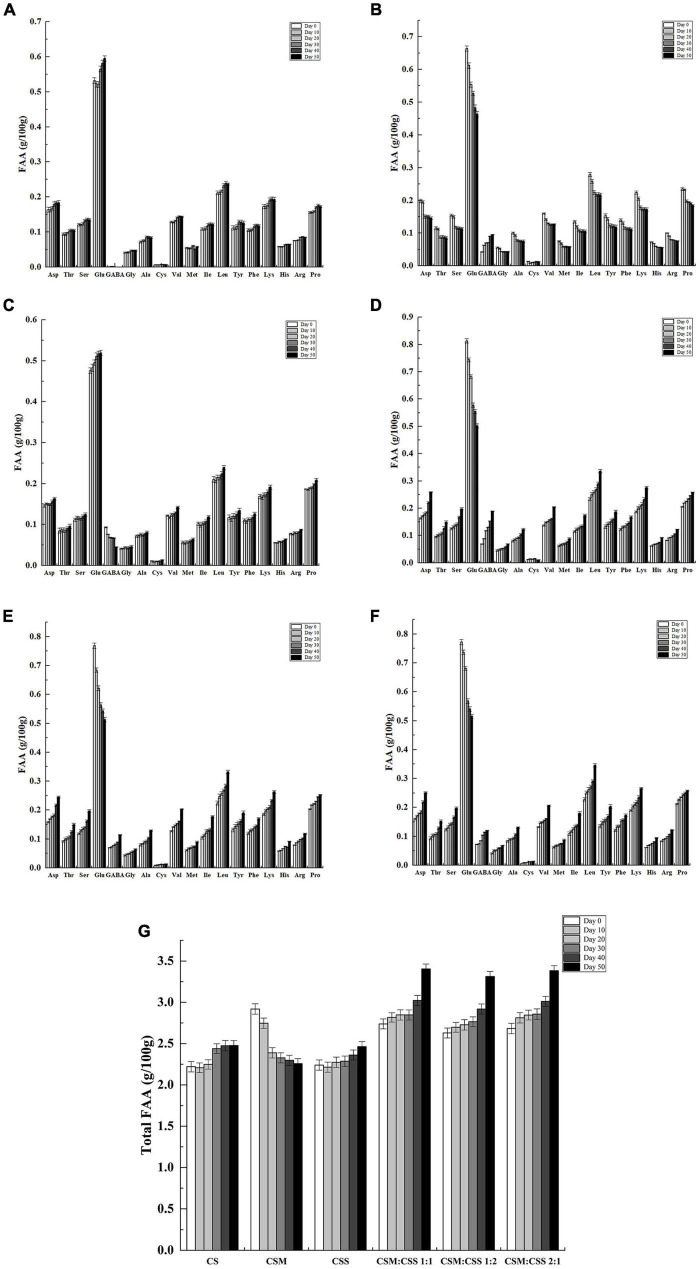
Free amino acid content of the cheeses (g/100 g). CS, CSM, CSS, CSM:CSS 1:1, CSM:CSS 1:2 and CSM:CSS 2:1 represent cheeses fermented using the commercial starter (Control group), *K. marxianus* B13–5, *S. cerevisiae* DL6–20, *K. marxianus* B13–5: *S. cerevisiae* DL6–20 1:1, *K. marxianus* B13–5: *S. cerevisiae* DL6–20 1: 2, and *K. marxianus* B13–5: *S. cerevisiae* DL6–20 2:1. **(A–F)** FAA content of CS, CSM, CSS, CSM:CSS 1:1, CSM:CSS 1:2 and CSM :CSS 2:1. **(G)** Represents the change in the total content of FAA in different cheeses. Error bars indicate standard deviations.

In general, the protein content of the six cheeses decreased during the ripening period, which is consistent with the results of Xu et al. (2020). The main reason is that during cheese ripening, protein is continuously broken down into peptides and amino acids under the action of proteases produced by microorganisms (Mane et al., 2019). In the late stage of cheese ripening, the protein content of CSM was the highest, although the protein content did not change much, the degree of proteolysis was small, and the total FAA content (Figure 3G) was less than that in the other groups. FAAs are the precursor of volatile aromatic compounds, which directly affect the taste and aroma of cheese (Murtaza et al., 2014). The protein content of CS and CSS changed significantly during the ripening period and the protein content was lower in the later stages. The protein content of mixed-strain fermented cheese was generally higher in the late stage of ripening, but the protein content of CSM:CSS 2:1 was not significantly increased. The protein content of CSM:CSS 1:1 decreased significantly during the ripening period, and the protein content was higher at 50 days. The CSM:CSS 1:1 group showed relatively well results in terms of amino acids and flavor substances (Figure 3 and Supplementary Table 1).

### Gamma Aminobutyric Acid Content and Amino Acid Content

Proteolysis was one of the most important biochemical reactions in the ripening process and had a great impact on the flavor and texture of most cheese varieties (Paul and Maria José, 2000). Amino acids were used as substrates for the production of flavor compounds (Kato et al., 1989). As shown in Figure 3, during the cheese ripening process, a total of 18 amino acids were detected, of which glutamic acid, leucine, lysine, proline and aspartic acid were present in the highest amounts and accounting for between 47 and 55% of the total amino acids. Glutamic acid and aspartic acid provide umami flavor (Kato et al., 1989), whereas proline and leucine have a bitter taste (Zhao et al., 2016). The glutamic acid contents in CSM, CSM:CSS 1:1, CSM:CSS 1:2 and CSM:CSS 2:1 showed a downward trend, while the GABA content increased. During the 50-day ripening process, the GABA content of CSM increased from 0.042 to 0.096 g/100 g, the content of CSM:CSS 1:1 increased from 0.069 to 0.189 g/100 g, the content of CSM:CSS 1:2 increased from 0.069 to 0.114 g/100 g, and the content of CSM:CSS 2:1 increased from 0.072 to 0.12 g/100 g, whereas GABA was almost undetected in the control group. The cheese in the CSM:CSS1:1 group had the highest GABA content in the later stage of ripening, reaching 0.189 g/100 g. GABA was not derived from casein, but was a product of microbial metabolism. It was mainly synthesized by decarboxylation of glutamate by glutamate decarboxylase. It has been reported that eating cheese containing GABA at 0.016 g/50 g lowered the blood pressure of the individuals by 3.5 mmHg (Pouliot-Mathieu et al., 2013). Consumption of fermented milk containing 0.02 g GABA has been shown to lower blood pressure (Diez-Gutiérrez et al., 2020). In this study, we show that this index was achieved in the cheeses in the CSM, CSM:CSS 1:1, CSM:CSS 1:2 and CSM:CSS 2:1 group. The total FAA concentration was often used to evaluate the flavor intensity of cheese at each ripening stage, and it can also be used as an indicator of cheese ripening (Bustamante et al., 2003). As shown in Figure 3G, the total FAA levels increased with ripening time in all the groups with the exception of the CSM group, in which the total FAA content decreased gradually during ripening. As shown in Figure 3G, the total FAA content of the cheeses in the experimental groups were higher than that in the control group may be explained the growth of both bacteria and yeast on the cheese surface leads to the production of proteolytic enzymes, which increase the FAA content (Sousa et al., 2001). In addition, the changes in the total FAA contents in cheeses fermented with mixed bacteria indicate a faster proteolysis rate than that associated with cheese prepared using a single bacterial strain (Niro et al., 2017). At the end of the ripening period, the total FAA contents in the three mixed-strain groups were similar. This may be due to inhibition of the proteolytic activity of microorganisms in the cheese by the increased levels of FFAs (especially short-chain FFAs; Hernández et al., 2009).

### Volatile Compound Contents

**FIGURE 4:**
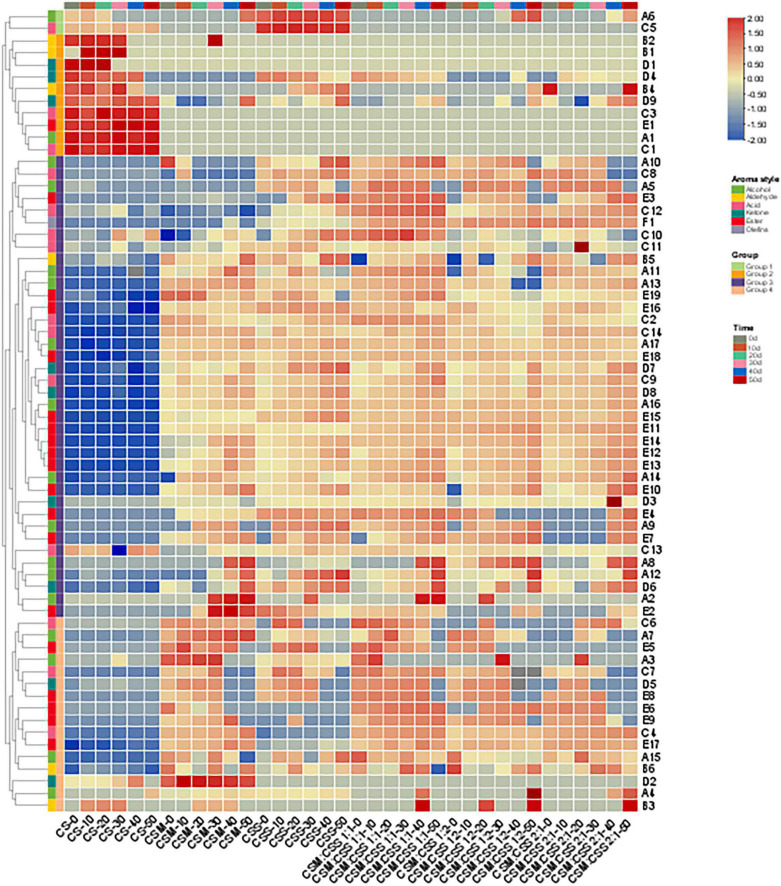
Heatmap analysis of the contents of volatile components in cheeses during six fermentation periods (0, 10, 20, 30, 40, and 50 days). CS, CSM, CSS, CSM:CSS 1:1, CSM:CSS 1:2 and CSM:CSS 2:1 represent cheeses fermented using the commercial starter (Control group), *K. marxianus* B13–5, *S. cerevisiae* DL6–20, *K. marxianus* B13–5: *S. cerevisiae* DL6–20 1:1, *K. marxianus* B13–5: *S. cerevisiae* DL6–20 1:2, and *K. marxianus* B13–5: *S. cerevisiae* DL6–20 2:1. (A1. 2-Furanmethanol; A2. 1-nonanol; A3. 1-Octanol; A4. 2,3-Butanediol; A5. 3-Methyl-1-pentanol; A6. 1-Pentanol; A7. 2-Pentanol; A8. 1-Hexanol; A9. 3-(Methylthio)-1-propanol; A10. 1-Mutanol; A11. 2-Nonanol; A12. 1,2-Propanediol; A13. 2-Methyl-1-propanol; A14. 2-Heptanol; A15. 3-Methyl-2-butanol; A16. Benzene ethanol; A17. Iso amyl alcohol; B1. 2-Furan-carboxaldehyde; B2. 2-Hexenal; B3. Octanal; B4. Hexanal; B5. Nonanal; B6. Acetaldehyde; C1. 2-Ethyl-butanoic acid; C2. 2-Oxo-propanoic acid; C3. Pentanoic acid; C4. 2-Methyl-butanoic acid; C5. Propanoic acid; C6. Heptanoic acid; C7. Nonanoic acid; C8. 3-Methyl-butanoic acid; C9. Decanoic acid; C10. Butanoic acid; C11. Hexanoic acid; C12. Octanoic acid; C13. Acetic acid; C14. 2-Methyl-propanoic acid; D1. 3-Heptanone; D2. Dihydro-2-methyl-3(2H)-furanone; D3. 2-Octanone; D4. 2-Pentanone; D5. 2-Undecanone; D6. 1-Hydroxy-2-propanone; D7. 2-Heptanone; D8. 2-Nonanone; D9. 3-Hydroxy-2-butanone; E1. Acetic acid, methyl ester; E2. Pentanoic acid, ethyl ester; E3. Butanoic acid, pentyl ester; E4. Acetic acid, 2- methyl-propyl ester; E5. 1,2-Benzene dicarboxylic acid-bis(2-methylpropyl) ester; E6. Acetic acid, propyl ester; E7. Butyrolactone; E8. 9-Decenoic acid, ethyl ester; E9. Nonanoic acid, ethyl ester; E10. 2-Hydroxy-Propanoic acid ethyl ester; E11. Butanoic acid, ethyl ester; E12. Heptanoic acid, ethyl ester; E13. Decanoic acid, ethyl ester; E14. Hexanoic acid, ethyl ester; E15. 1-Butanol, 3- methyl-, acetate; E16. Acetic acid, butyl ester; E17. Acetic acid, 2-phenylethyl ester; E18. Octanoic acid, ethyl ester; E19. Acetic acid ethyl ester; F1. Benzene ethenyl).

Gamma Aminobutyric Acid-producing yeast strains are beneficial to the production of industrial cheese. Our analysis of volatile components produced during the ripening are shown in Supplementary Table 1 and Figure 4. A total of 66 different flavor substances (17 alcohols, 6 aldehydes, 14 acids, 9 ketones, 19 esters and 1 olefin) were identified, although the content varied with the type and proportion of added yeast.

Four groups of volatile components were identified (groups 1–4, containing 2, 10, 38 and 16 aromatic compounds, respectively). The contents of the flavor components of the second group in CS (2-hexenal, 2-furan-carboxaldehyde, 3-heptanone, 2-pentanone, hexanal, 3-hydroxy-2-butanone, pentanoic acid, acetic acid, methyl ester, 2-furanmethanol, and 2-ethyl-butanoic acid) were significantly higher than those in the experimental groups, while the contents of the flavor components of groups 3 and 4 were lower than those in the experimental groups. Aldehydes are derived mainly from the catabolism of FAAs (Fox et al., 2017; Ganesan and Weimer, 2017) and are converted into corresponding alcohols by alcohol dehydrogenase, or oxidized into corresponding carboxylic acids by aldehyde dehydrogenase (Yvon and Rijnen, 2001; Fox et al., 2017; Ganesan and Weimer, 2017). The biosynthesis of primary and aromatic alcohols and carboxylic acids is mainly attributed to the metabolism of yeast. Ketones can also be further converted into secondary alcohols by reductases (Collins et al., 2003; Fox et al., 2017). These substances give cheese a distinctive flavor. Acetic acid, methyl ester gives cheese an aromatic taste, and 2-ethyl-butanoic acid confers a sour taste. CSM:CSS 1:1, CSM:CSS 1:2 and CSM:CSS 2:1 produced higher levels of the esters in the fourth group of flavor components (acetic acid, propyl ester, 9-decenoic acid, ethyl ester, nonanoic acid, ethyl ester, acetic acid, 2-phenylethyl ester) and aldehydes (octanal) than those in the CSM and CSS groups fermented by a single bacterial strain. These results are related to the esterification of FFAs and alcoholysis in cheese (Alewijn et al., 2005; Bertuzzi et al., 2017). Esterification is the formation of esters from alcohols and carboxylic acids by esterases, while alcoholysis is the formation of esters from alcohols and acylglycerols or acyl-CoA (derived from the metabolism of FAA and/or carbohydrates) by acyltransferases. In alcoholysis, the fatty acyl groups of acylglycerol and acyl-CoA derivatives are transferred directly to the alcohol (Liu et al., 2004). Some esters are characterized by a low perception threshold and are generally appreciated for their sweet, fruity and floral aromas, as well as their ability to minimize the clarity and bitterness of cheese (Curioni and Bosset, 2002; Liu et al., 2004). This phenomenon is reflected in the decrease in the content of the first group of flavor substances (3-methyl-1-pentanol, propanoic acid) in the mixed-strain fermented cheese. These substances give cheese a distinctive flavor. Acetic acid, propyl ester is responsible for a soft fruity flavor, while nonanoic acid, ethyl ester provides oil, fruit and brandy-like aromas and acetic acid, 2-phenylethyl ester produces a rose-like aroma. Octanal has a strong fruity aroma, with a pleasant orange aroma when the content at low levels (Juan et al., 2007). CSM fermented by single bacteria, we detected higher levels of alcohols (1-octanol) and ketones (dihydro-2-methyl-3(2H)-Furanone) in the group 4 components in the CSS group, and higher levels of alcohols in group 3 (1-hexanol, 1,2-propanediol, 1-nonanol) compared with those in the mixed fermentation of CSM:CSS 1:1, CSM:CSS 1:2 and CSM:CSS 2:1 groups. 1-Nonanol gives cheese a pleasant aroma of rose and orange, while 1-hexanol gives the cheese a light green leafy aroma, with a hint of wine, fruit and fatty aromas and 1-octanol confers a citrus, orange peel and rose-like aroma (Curioni and Bosset, 2002). These results indicate that single-strain fermentation is conducive to the production of alcohols, while mixed-strain fermentation produces more esters.

### Multivariate Statistical Analysis of Cheese Flavor Compounds During Storage

**FIGURE 5:**
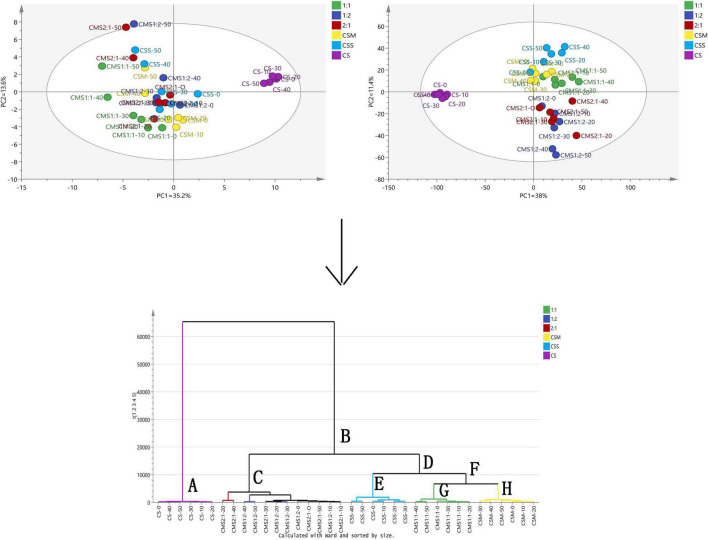
Principal component analysis, orthogonal partial least squares discriminant analysis and hierarchical cluster analysis (HCA) of volatile components in cheese. The left and right figures represent the PCA and OPLS-DA results of different processed cheese samples, and the bottom figure represents the HCA results. CS, CSM, CSS, CSM:CSS 1:1, CSM:CSS 1:2 and CSM:CSS 2:1 indicate the use of the commercial starter (Control group), *K. marxianus* B13–5, *S. cerevisiae* DL6–20, *K. marxianus* B13–5: *S. cerevisiae* DL6–20 1:1, *K. marxianus* B13–5: *S. cerevisiae* DL6–20 1: 2, and *K. marxianus* B13–5: *S. cerevisiae* DL6–20 2:1.

We conducted multivariate analysis to explore the compound changes of different types and ratios of yeast-fermented cheese samples during storage. Principal component analysis (PCA) and OPLS-DA were used to group and cluster the samples. PCA is a statistical analysis that decomposes a data set into orthogonal components, and its linear combination (principal components) approximates the original data to any required accuracy. In most cases, two principal components are sufficient to explain a large part of the changes in the original parameters (Delgado et al., 2011). OPLS-DA divides the systematic variation of the X area into two model parts: the first part simulates the co-variation between X and Y, and the second part captures the systematic variation of the X area unrelated to Y (Shiota et al., 2015). As shown in Figure 5, the six groups of samples were found to be evenly separated in the OPLS-DA charts. Cheese samples with different types and proportions of yeast could be divided roughly into two groups. In the PCA, PC1 and PC2 were 35.6 and 13.6%, respectively, and the sum of PC1 and PC2 was 48.8%. Compared with the PCA, OPLS-DA showed a better separation effect on the six groups of samples. The cross-validation predictive ability obtained by the model was Q2(Y) = 0.63, and the total explained variance was R2(X) = 0.907, R2(Y) = 1.

As shown in Figure 5, the HCA revealed that the samples could be divided into two categories: control group (CS) and experimental groups (CSS, CSM, CSM:CSS 1:1, CSM:CSS 1:2 and CSM:CSS 2:1). This analysis showed that yeast treatments have a clearly distinctive aroma profile from those containing only LAB. To facilitate the discussion, the first group is designated A, and the second group B. In order to further study the influence of the different types and proportions of yeasts on cheese fermentation, we divided group B into groups C and D according to the results of the HCA. Group D was then divided into groups E and F and group F we divided into groups G and H.

**FIGURE 6:**
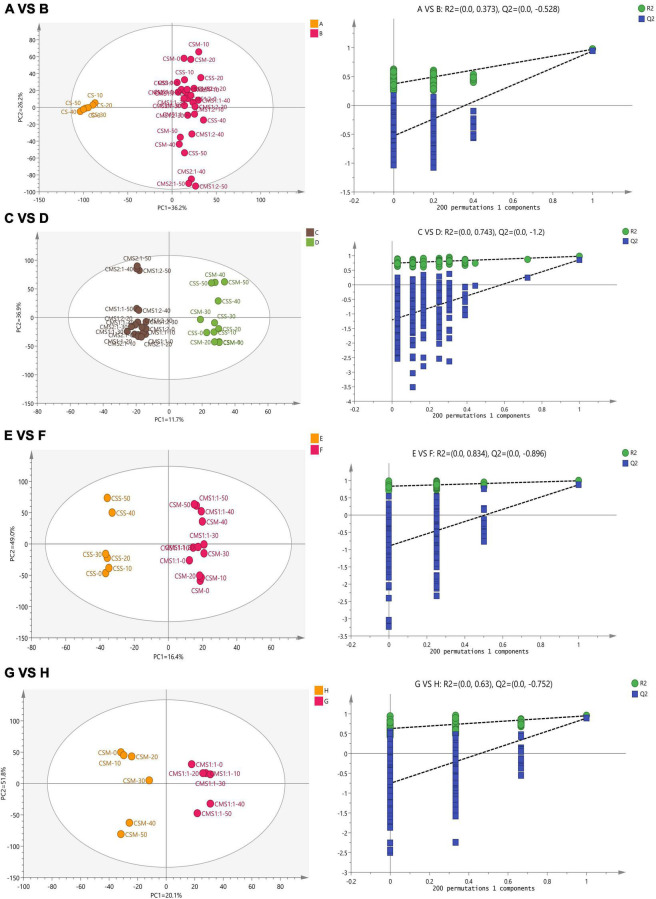
Multivariate statistical analysis of flavor compounds in cheese during storage based on HCA. The results of discriminant analysis of group A versus group B, group C versus group D, group E versus group F, and group G versus group H from top to bottom. CS, CSM, CSS, CSM:CSS 1:1, CSM:CSS 1:2 and CSM:CSS 2:1 indicate the use of the commercial starter (Control group), *K. marxianus* B13–5, *S. cerevisiae* DL6–20, *K. marxianus* B13–5: *S. cerevisiae* DL6–20 1:1, *K. marxianus* B13–5: *S. cerevisiae* DL6–20 1: 2, and *K. marxianus* B13–5: *S. cerevisiae* DL6–20 2:1.

**FIGURE 7:**
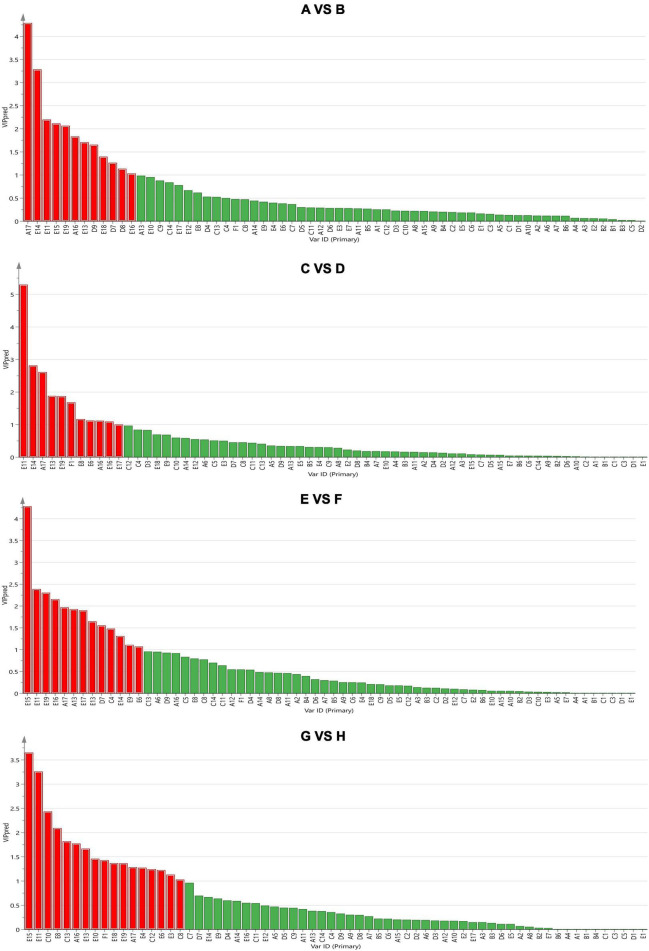
The importance of variables in the projection (VIP = 6. VIP > 1 indicates extreme importance). The comparison between group A and group B, group C and group D, group E and group F, group G and group H from top to bottom. CS, CSM, CSS, CSM:CSS 1:1, CSM:CSS 1:2 and CSM:CSS 2:1 indicate the use of the commercial starter (Control group), *K. marxianus* B13–5, *S. cerevisiae* DL6–20, *K. marxianus* B13–5: *S. cerevisiae* DL6–20 1:1, *K. marxianus* B13–5: *S. cerevisiae* DL6–20 1: 2, and *K. marxianus* B13–5: *S. cerevisiae* DL6–20 2:1. (A1. 2-Furanmethanol; A2. 1-nonanol; A3. 1-Octanol; A4. 2,3-Butanediol; A5. 3-Methyl-1-pentanol; A6. 1-Pentanol; A7. 2-Pentanol; A8. 1-Hexanol; A9. 3-(Methylthio)-1-propanol; A10. 1-Mutanol; A11. 2-Nonanol; A12. 1,2-Propanediol; A13. 2-Methyl-1-propanol; A14. 2-Heptanol; A15. 3-Methyl-2-butanol; A16. Benzene ethanol; A17. Iso amyl alcohol; B1. 2-Furan-carboxaldehyde; B2. 2-Hexenal; B3. Octanal; B4. Hexanal; B5. Nonanal; B6. Acetaldehyde; C1. 2-Ethyl-butanoic acid; C2. 2-Oxo-propanoic acid; C3. Pentanoic acid; C4. 2-Methyl-butanoic acid; C5. Propanoic acid; C6. Heptanoic acid; C7. Nonanoic acid; C8. 3-Methyl-butanoic acid; C9. Decanoic acid; C10. Butanoic acid; C11. Hexanoic acid; C12. Octanoic acid; C13. Acetic acid; C14. 2-Methyl-propanoic acid; D1. 3-Heptanone; D2. Dihydro-2-methyl-3(2H)-furanone; D3. 2-Octanone; D4. 2-Pentanone; D5. 2-Undecanone; D6. 1-Hydroxy-2-propanone; D7. 2-Heptanone; D8. 2-Nonanone; D9. 3-Hydroxy-2-butanone; E1. Acetic acid, methyl ester; E2. Pentanoic acid, ethyl ester; E3. Butanoic acid, pentyl ester; E4. Acetic acid, 2- methyl-propyl ester; E5. 1,2-Benzene dicarboxylic acid-bis(2-methylpropyl) ester; E6. Acetic acid, propyl ester; E7. Butyrolactone; E8. 9-Decenoic acid, ethyl ester; E9. Nonanoic acid, ethyl ester; E10. 2-Hydroxy-Propanoic acid ethyl ester; E11. Butanoic acid, ethyl ester; E12. Heptanoic acid, ethyl ester; E13. Decanoic acid, ethyl ester; E14. Hexanoic acid, ethyl ester; E15. 1-Butanol, 3- methyl-, acetate; E16. Acetic acid, butyl ester; E17. Acetic acid, 2-phenylethyl ester; E18. Octanoic acid, ethyl ester; E19. Acetic acid ethyl ester; F1. Benzene ethenyl).

Subsequently, the supervised orthogonal projection method was applied again to the OPLS-DA to identify the differences in flavor substances between A and B, C and D, E and F, and G and H. A shown in Figure 6, the two groups were clearly separated in the score chart, where R2(x) is 0.951, Q2(x) is 0.89; R2(x) is 0.979, Q2(x) is 0.861; R2(x) is 0.992, Q2(x) is 0.876; R2(x) is 0.972, Q2(x) is 0.943, indicating the validity of the constructed model. Figure 7 shows the VIP diagram of the OPLS-DA model. Flavor compounds with a VIP greater than 1.0 in the OPLS-DA model were identified as substances that showed major differences.

As shown in Figure 7, the different substances identified in the comparison of A (control group) and B (experimental group) were A17 (isoamyl alcohol), E14 (hexanoic acid, ethyl ester), E11 (butanoic acid, ethyl ester), E15 (1-butanol, 3- methyl-, acetate), E19 (acetic acid ethyl ester), A16 (Benzene ethanol), E13 (Decanoic acid, ethyl ester), D9 (3-hydroxy-2-Butanone), E18 (octanoic acid, ethyl ester), D7 (2-heptanone), and D8 (2-nonanone), and E16 (Acetic acid, butyl ester). Compared with the control group, the cheese made by adding yeast produces higher levels of esters, alcohols and ketones, giving the cheese a more fruity and wine-like scent.

As shown in Figure 7, the different substances identified in the comparison of C (CSM:CSS 1:2 and CSM:CSS 2:1) and D (CSS, CSM, CSM:CSS 1:1) were E11 (butanoic acid, ethyl ester), E14 (hexanoic acid, ethyl ester), A17 (isoamyl alcohol), E13 (decanoic acid, ethyl ester), E19 (acetic acid ethyl ester), F1 (benzene ethenyl), E8 (9-decenoic acid, ethyl ester), E6 (acetic acid, propyl ester), A16 (benzene ethanol), E16 (acetic acid, butyl ester), and E17 (acetic acid, 2-phenylethyl ester). The mixed fermentation using CSM:CSS 1:2 and CSM:CSS 2:1 produces more esters than the fermentation using either CSS and CSM alone or mixed fermentation using CSM:CSS 1:1. These esters have a fruity aroma, indicating that *K. marxianus* B13–5 and *S. cerevisiae* DL6–20 have good aroma-producing properties when mixed at 1:2 and 2:1.

As shown in Figure 7, the different substances identified in the comparison of E (CSS) and F (CSM, CSM: CSS 1:1) were E15 (1-butanol, 3- methyl-, acetate), E11 (butanoic acid, ethyl ester), E19 (acetic acid ethyl ester), E16 (acetic acid, butyl ester), A17 (isoamyl alcohol), A13 (2-methyl-1-propanol), E17 (acetic acid, 2-phenylethyl ester), E13 (decanoic acid, ethyl ester), D7 (2-heptanone), C4 (2-methyl-butanoic acid), E14 (hexanoic acid, ethyl ester), E9 (nonanoic acid, ethyl ester), and E6 (acetic acid, propyl ester). CSM, CSM: CSS 1:1 produces more esters than CSS, which produces more alcohol, and has a stronger wine aroma.

As shown in Figure 7, the different substances identified in the comparison of G (CSM:CSS 1:1) and H (CSM) were E15 (1-butanol, 3- methyl-, acetate), E11 (butanoic acid, ethyl ester), C10 (Butanoic acid), E8 (9-Decenoic acid, ethyl ester), C13 (Acetic acid), A16 (benzene ethanol), E13 (decanoic acid, ethyl ester), E10 (2-hydroxy-propanoic acid ethyl ester), F1 (benzene ethenyl), E18 (octanoic acid, ethyl ester), E19 (acetic acid ethyl ester), A17 (isoamyl alcohol), E4 (acetic acid, 2- methyl-propyl ester), C12 (octanoic acid), E6 (acetic acid, propyl ester), E3 (butanoic acid, pentyl ester), and C8 (3-methyl-butanoic acid). CSM:CSS 1:1 produces more esters and acids than CSM. Butyric acid and acetic acid have a pungent odor, and caprylic acid has a slightly unpleasant odor and burnt odor, although a fruity aroma is obtained by dilution. These results show that when *K. marxianus* B13–5 and *S. cerevisiae* DL6–20 are mixed at a ratio of 1:1, they have a distinct aroma-producing performance and fermentation potential. Therefore, mixed fermentation is conducive to the production of cheese aroma, and CSM:CSS 1:2 and CSM:CSS 2:1 produce stronger aromas.

## Conclusion

This study provides further clarification of the effect of the addition ratio of GABA-producing *S. cerevisiae* DL6–20 and *K. marxianus* B13–5 on cheese quality. The results showed that the total FAA content of cheeses produced by mixed-strain fermentation was higher than that of cheese produced by the individual strains. The GABA contents of CSM:CSS 1:2, CSM:CSS, and CSM:CSS1 :1 were 0.114 g/100 g, 0.12 g/100 g, and 0.189 g/100 g, respectively. The highest GABA production occurs in the late maturation period, reaching 189 mg/100 g. CSM:CSS 1:1 has a higher number of LAB and yeasts than other cheeses. In addition, CSM:CSS 1:1 also contributes to the cheese texture. The protein content of mixed-strain fermented cheese is generally high in the late stage of ripening, especially the protein content of CSM:CSS 1:1 during the maturation period, and the protein content is higher at 50 days, and the CSM:CSS 1:1 moisture content is low, which is easy to save. With the exception of water and protein contents, there were no significant differences between the groups in terms of the other physical and chemical indicators. In general, the mixed-strain fermentation is more beneficial to the production of cheese flavor substances than the single-strain fermentation, and the GABA content is higher. Although the volatile component content of CSM:CSS 1:1 was lower than that of CSM:CSS 1:2, CSM:CSS 2:1, its high GABA content and low moisture content is more conducive to storage and the industrial production.

## Data Availability Statement

The original contributions presented in the study are included in the article/Supplementary Material, further inquiries can be directed to the corresponding author.

## Author Contributions

SL, YZ, and BL contributed to conception and design of the study. PY organized the database. YZ performed the statistical analysis. SL wrote the first draft of the manuscript. XL, TW, YL, KZ, HS, SLL, HJ, and ZF wrote sections of the manuscript. All authors contributed to manuscript revision, read, and approved the submitted version.

## Conflict of Interest

SL was employed by Henan Shuanghui Investment & Development Co., Ltd. XL was employed by Guangdong Yikewei Biotech Co., Ltd. The remaining authors declare that the research was conducted in the absence of any commercial or financial relationships that could be construed as a potential conflict of interest.

## Publisher’s Note

All claims expressed in this article are solely those of the authors and do not necessarily represent those of their affiliated organizations, or those of the publisher, the editors and the reviewers. Any product that may be evaluated in this article, or claim that may be made by its manufacturer, is not guaranteed or endorsed by the publisher.
