# Supplementary material for: The Effect of the Ratio of Gamma Aminobutyric Acid-Producing Saccharomyces cerevisiae DL6–20 and Kluyveromyces marxianus B13–5 Addition on Cheese Quality
Source: Front Microbiol. 2022 Jun 23;13:900394. doi: 10.3389/fmicb.2022.900394 (PMC9260010; doi:10.3389/fmicb.2022.900394)
Supplement: Supplementary file 1 [file Table_1.DOCX]

**Supplementary Table 1** The content of the aroma components in the cheeses.

|  |  |  | CS | | | | | |
| --- | --- | --- | --- | --- | --- | --- | --- | --- |
|  | compound | RI | CS-0 | CS-10 | CS-20 | CS-30 | CS-40 | CS-50 |
| A1 | 2-Furanmethanol | 1660 | 5.698±0.561^a^ | 4.607±0.492^a^ | 4.724±0.421^a^ | 6.221±0.613^a^ | 4.516±0.531^a^ | 5.669±0.448^a^ |
| A2 | 1-nonanol | 1660 | 0 | 0 | 0 | 0 | 0 | 0 |
| A3 | 1-Octanol | 1557 | 0 | 0 | 0 | 0.842±0.082^c^ | 0 | 0 |
| A4 | 2,3-Butanediol | 1543 | 0.000 | 0 | 0 | 0 | 0 | 1.15±0.154^b^ |
| A5 | 3-methyl-1-Pentanol | - | 0.47±0.041^d^ | 0.527±0.047^d^ | 0 | 0 | 0 | 0 |
| A6 | 1-Pentanol | 1250 | 3.291±0.366^b^ | 4.516±0.489^b^ | 2.761±0.313^b^ | 0 | 0 | 0 |
| A7 | 2-Pentanol | 1119 | 0 | 0 | 0 | 0 | 0 | 0.157±0.02^b^ |
| A8 | 1-Hexanol | 1355 | 0 | 0 | 0 | 0 | 0 | 0 |
| A9 | 3-(methylthio)-1-Propanol | 1719 | 0 | 0 | 0 | 0 | 0 | 0 |
| A10 | 1-Butanol | 1142 | 0 | 0 | 0 | 0 | 0 | 0 |
| A11 | 2-Nonanol | 1521 | 0 | 0 | 0 | 0 | 0 | 0 |
| A12 | 1,2-Propanediol | 1600 | 0 | 1.42±0.169^c^ | 0.823±0.089^d^ | 0.085±0.003^d^ | 0.008±0.001^e^ | 0.082±0.001^e^ |
| A13 | 2-methyl-1-Propanol | - | 0 | 0 | 0 | 0 | 0 | 0 |
| A14 | 2-Heptanol | 1320 | 0 | 0.235±0.015^c^ | 0.649±0.056^d^ | 0.542±0.045^c^ | 0.483±0.039^d^ | 0.411±0.032^d^ |
| A15 | 3-methyl- 2-Butanol | - | 0 | 0 | 0.213±0.015^e^ | 0.095±0.004^e^ | 0.086±0.003^d^ | 0.15±0.009^d^ |
| A16 | Benzeneethanol | 1906 | 0.614±0.012^e^ | 0.355±0.023^d^ | 0.368±0.027^d^ | 0.375±0.043^d^ | 0 | 0 |
| A17 | ISO AMYL ALCOHOL | 1209 | 4.117±0.386^c^ | 1.306±0.105^c^ | 3.248±0.299^d^ | 4.244±0.398^d^ | 4.789±0.453^d^ | 5.644±0.538^c^ |
| B1 | 2-furan-carboxaldehyde | 1461 | 0.000 | 0.421±0.025^a^ | 0.486±0.037^a^ | 0.509±0.042^a^ | 0 | 0 |
| B2 | 2-Hexenal | 1090 | 0.996±0.087^a^ | 1.108±0.103^a^ | 1.068±0.103^a^ | 1.009±0.103^b^ | 0 | 0 |
| B3 | Octanal | 1289 | 0.000 | 2.002±0.218^a^ | 2.282±0.246^b^ | 2.362±0.254^a^ | 0 | 0 |
| B4 | Hexanal | 1083 | 18.39±1.166^b^ | 26.602±1.987^a^ | 9.816±0.909^a^ | 34.723±2.799^a^ | 3.625±0.29^b^ | 0 |
| B5 | Nonanal | 1391 | 2.827±0.245^d^ | 1.793±0.141^d^ | 4.751±0.437^d^ | 3.728±0.335^d^ | 1.661±0.128^e^ | 0.993±0.058^d^ |
| B6 | Acetaldehyde | 702 | 0.000 | 0.633±0.044^c^ | 0.491±0.012^e^ | 0.382±0.019^d^ | 0.258±0.007^d^ | 0 |
| C1 | 2-ethyl-Butanoic acid | - | 1.606±0.138^a^ | 1.197±0.097^a^ | 1.207±0.098^a^ | 1.499±0.127^a^ | 1.505±0.128^a^ | 1.518±0.129^a^ |
| C2 | 2-oxo-Propanoic acid | - | 0.000 | 0 | 0 | 0 | 0 | 0 |
| C3 | Pentanoic acid | 1733 | 2.075±0.225^a^ | 1.414±0.158^a^ | 3.14±0.331^a^ | 1.978±0.215^a^ | 1.971±0.214^a^ | 1.81±0.198^a^ |
| C4 | 2-methyl-Butanoic acid | - | 0.000 | 0 | 0 | 0 | 0 | 0 |
| C5 | Propanoic acid | 1535 | 2.403±0.228^b^ | 2.644±0.252^b^ | 1.916±0.18^b^ | 2.893±0.277^b^ | 2.09±0.197^b^ | 2.71±0.259^b^ |
| C6 | Heptanoic acid | 1950 | 0.655±0.043^c^ | 0.708±0.048^c^ | 0.753±0.052^e^ | 0.927±0.07^d^ | 0.703±0.047^c^ | 0.685±0.046^c^ |
| C7 | Nonanoic acid | 2171 | 0.000 | 0 | 0 | 0 | 0 | 0 |
| C8 | 3-methyl-Butanoic acid | - | 1.157±0.101^d^ | 1.208±0.106^d^ | 1.353±0.12^c^ | 1.392±0.124^c^ | 1.428±0.128^d^ | 1.739±0.159^d^ |
| C9 | Decanoic acid | 1097 | 2.964±0.323^d^ | 2.006±0.228^d^ | 3.806±0.408^d^ | 4.169±0.444^d^ | 1.45±0.172^c^ | 2.599±0.287^d^ |
| C10 | Butanoic acid | 1625 | 124.433±9.071^cd^ | 135.996±10.228^c^ | 115.045±8.133^c^ | 231.784±19.806^b^ | 162.186±12.847^c^ | 228.511±19.479^b^ |
| C11 | Hexanoic acid | 1846 | 68.938±5.87^b^ | 58.684±4.844^b^ | 58.701±4.846^b^ | 83.956±7.372^b^ | 52.772±4.253^c^ | 70.786±6.055^b^ |
| C12 | Octanoic acid | 2060 | 17.001±1.176^d^ | 14.895±0.966^d^ | 17.887±1.265^d^ | 22.2±1.696^d^ | 10.851±0.561^e^ | 15.375±1.014^c^ |
| C13 | Acetic acid | 1449 | 263.781±25.354^a^ | 205.975±19.574^a^ | 226.253±21.601^a^ | 0.086±0.003^d^ | 318.93±30.869^a^ | 270.987±26.075^a^ |
| C14 | 2-methyl-Propanoic acid | - | 2.612±0.232^d^ | 1.774±0.148^d^ | 2.196±0.191^d^ | 2.404±0.211^d^ | 2.792±0.25^d^ | 3.74±0.345^d^ |
| D1 | 3-Heptanone | 1161 | 2.894±0.258^a^ | 4.682±0.437^a^ | 6.338±0.603^a^ | 0 | 0 | 0 |
| D2 | dihydro-2-methyl-3(2H)-Furanone | - | 0.146±0.012^b^ | 0.158±0.013^b^ | 0.165±0.014^b^ | 0.265±0.024^b^ | 0.52±0.049^b^ | 0 |
| D3 | 2-Octanone | 1287 | 0.000 | 0 | 0 | 0 | 0 | 0 |
| D4 | 2-Pentanone | 981 | 73.891±6.065^a^ | 48.474±4.323^a^ | 33.62±2.838^a^ | 34.825±2.959^a^ | 31.057±2.582^a^ | 0 |
| D5 | 2-Undecanone | 1598 | 0.000 | 0.911±0.003^d^ | 1.027±0.064^c^ | 1.462±0.039^c^ | 0.675±0.042^b^ | 0.783±0.063^a^ |
| D6 | 1-hydroxy-2-Propanone | - | 0.554±0.064^f^ | 1.167±0.126^d^ | 0.855±0.095^e^ | 1.035±0.113^e^ | 0.472±0.056^d^ | 0.234±0.032^d^ |
| D7 | 2-Heptanone | 1182 | 26.533±1.67^c^ | 28.501±1.867^c^ | 34.692±1.486^d^ | 44.752±2.492^e^ | 19.618±0.979^d^ | 33.543±2.371^d^ |
| D8 | 2-Nonanone | 1390 | 5.001±0.442^d^ | 5.905±0.533^b^ | 5.55±0.497^d^ | 9.693±0.911^c^ | 4.877±0.43^d^ | 6.358±0.578^c^ |
| D9 | 3-hydroxy-2-Butanone | - | 460.566±40.075^a^ | 366.189±32.338^a^ | 379.338±33.653^a^ | 478.911±41.909^a^ | 484.254±42.443^a^ | 441.386±38.157^a^ |
| E1 | ACETIC ACID, METHYL ESTER | 828 | 1.822±0.124^a^ | 1.806±0.123^a^ | 1.782±0.12^a^ | 2.874±0.229^a^ | 2.675±0.21^a^ | 2.413±0.183^a^ |
| E2 | Pentanoic acid, ethyl ester | 1134 | 0 | 0 | 0 | 0 | 0 | 0 |
| E3 | Butanoic acid, pentyl ester | 1305 | 0 | 0 | 0 | 0 | 0 | 0 |
| E4 | acetic acid, 2- methyl-propyl ester | 1012 | 0 | 0 | 0 | 0 | 0 | 0 |
| E5 | 1,2-Benzenedicarboxylic acid-bis(2-methylpropyl) ester | 2536 | 0 | 0 | 0 | 0 | 0 | 0 |
| E6 | Acetic acid, propyl ester | 973 | 0 | 0 | 0 | 0 | 0 | 0 |
| E7 | Butyrolactone | 1632 | 0 | 0 | 0 | 0 | 0 | 0 |
| E8 | 9-Decenoic acid, ethyl ester | 1694 | 0 | 0 | 0 | 0 | 0 | 0 |
| E9 | Nonanoic acid, ethyl ester | 1531 | 0 | 0 | 0 | 0 | 0 | 0 |
| E10 | 2-hydroxy-Propanoic acid ethyl ester | 1347 | 0 | 0 | 0 | 0 | 0 | 0 |
| E11 | Butanoic acid, ethyl ester | 1035 | 0 | 0 | 0 | 0 | 0 | 0 |
| E12 | Heptanoic acid, ethyl ester | 1331 | 0 | 0 | 0 | 0 | 0 | 0 |
| E13 | Decanoic acid, ethyl ester | 1638 | 0 | 0 | 0 | 0 | 0 | 0 |
| E14 | Hexanoic acid, ethyl ester | 1233 | 0 | 0 | 0 | 0 | 0 | 0 |
| E15 | 1-Butanol, 3-methyl-, acetate | 1122 | 0 | 0 | 0 | 0 | 0 | 0 |
| E16 | Acetic acid, butyl ester | 1074 | 1.669±0.109^e^ | 3.194±0.261^d^ | 4.79±0.421^e^ | 5.36±0.478^e^ | 0 | 0 |
| E17 | Acetic acid, 2-phenylethyl ester | 1813 | 0 | 1.094±0.051^e^ | 0.848±0.027^d^ | 1.572±0.099^d^ | 2.58±0.2^e^ | 2.118±0.154^f^ |
| E18 | Octanoic acid, ethyl ester | 1435 | 1.293±0.071^c^ | 0.911±0.033^e^ | 0.282±0.02^c^ | 0.843±0.026^d^ | 1.883±0.13^d^ | 3.935±0.336^d^ |
| E19 | Acetic acid ethyl ester | 888 | 176.573±15.566^e^ | 187.889±16.698^e^ | 130.462±10.955^e^ | 110.644±8.973^d^ | 90.834±7.844^d^ | 84.946±6.921^f^ |
| F1 | Benzene ethenyl | 1261 | 0 | 0 | 0 | 0 | 0 | 0 |

|  |  |  | CSM | | | | | |
| --- | --- | --- | --- | --- | --- | --- | --- | --- |
|  | compound | RI | CSM-0 | CSM-10 | CSM-20 | CSM-30 | CSM-40 | CSM-50 |
| A1 | 2-Furanmethanol | 1660 | 0 | 0 | 0 | 0 | 0 | 0 |
| A2 | 1-nonanol | 1660 | 0 | 0 | 0 | 18.239±1.832^a^ | 29.446±2.462^a^ | 36.244±3.271^a^ |
| A3 | 1-Octanol | 1557 | 4.072±0.421^a^ | 4.284±0.472^b^ | 5.104±0.521^a^ | 5.587±0.528^b^ | 0 | 0 |
| A4 | 2,3-Butanediol | 1543 | 0.318±0.041^b^ | 0.325±0.042^b^ | 0.583±0.067^a^ | 0.646±0.074^a^ | 0.856±0.095^c^ | 0.701±0.079^b^ |
| A5 | 3-methyl-1-Pentanol | - | 0 | 0.255±0.02^d^ | 0 | 0 | 0 | 0 |
| A6 | 1-Pentanol | 1250 | 0 | 0 | 0 | 0 | 0 | 11.804±1.277^b^ |
| A7 | 2-Pentanol | 1119 | 3.125±0.285^b^ | 5.249±0.497^a^ | 6.138±0.586^b^ | 7.139±0.686^a^ | 8.245±0.797^a^ | 9.106±0.883^a^ |
| A8 | 1-Hexanol | 1355 | 0 | 0 | 0 | 0 | 33.115±3.042^b^ | 59.454±5.675^a^ |
| A9 | 3-(methylthio)-1-Propanol | 1719 | 0 | 0 | 6.937±0.657^b^ | 7.382±0.701^b^ | 7.705±0.734^c^ | 0 |
| A10 | 1-Butanol | 1142 | 7.908±0.827^a^ | 1.782±0.214^b^ | 0 | 0 | 0 | 0 |
| A11 | 2-Nonanol | 1521 | 7.625±0.795^c^ | 4.718±0.504^d^ | 8.273±0.859^c^ | 12.231±1.255^b^ | 32.601±3.292^a^ | 16.981±1.73^b^ |
| A12 | 1,2-Propanediol | 1600 | 0 | 0 | 0 | 4.425±0.47^c^ | 16.505±1.678^b^ | 46.839±3.657^d^ |
| A13 | 2-methyl-1-Propanol | - | 140.647±11.092^a^ | 174.726±14.5^a^ | 164.848±13.512^a^ | 195.331±16.56^a^ | 188.664±15.893^a^ | 281.795±25.207^a^ |
| A14 | 2-Heptanol | 1320 | 0 | 22.965±2.328^a^ | 27.472±2.778^ab^ | 30.018±3.033^a^ | 22.284±2.259^b^ | 16.358±1.667^c^ |
| A15 | 3-methyl- 2-Butanol | - | 11.168±1.074^c^ | 8.773±0.834^a^ | 2.536±0.211^d^ | 12.391±1.196^a^ | 5.198±0.477^b^ | 0 |
| A16 | Benzeneethanol | 1906 | 125.43±10.157^d^ | 165.732±14.187^c^ | 185.462±16.16^c^ | 247.062±22.32^c^ | 280.879±25.702^c^ | 311.266±28.741^b^ |
| A17 | ISO AMYL ALCOHOL | 1209 | 1305.76±86.594^b^ | 1482.563±104.274^b^ | 1425.954±98.613^c^ | 1549.56±110.974^b^ | 1855.888±141.607^b^ | 3220.246±278.043^a^ |
| B1 | 2-furan-carboxaldehyde | 1461 | 0 | 0 | 0 | 0 | 0 | 0 |
| B2 | 2-Hexenal | 1090 | 0 | 0 | 0 | 2.657±0.243^a^ | 0 | 0 |
| B3 | Octanal | 1289 | 0 | 0 | 1.376±0.156^c^ | 1.375±0.156^b^ | 1.372±0.155^b^ | 0 |
| B4 | Hexanal | 1083 | 0 | 0 | 0 | 0 | 0 | 0 |
| B5 | Nonanal | 1391 | 18.445±1.353^a^ | 10.734±0.581^c^ | 10.594±0.567^b^ | 9.936±0.956^b^ | 9.525±0.915^d^ | 31.504±3.112^a^ |
| B6 | Acetaldehyde | 702 | 3.928±0.374^b^ | 1.739±0.155^a^ | 0.978±0.056^d^ | 3.494±0.33^b^ | 0.872±0.068^c^ | 3.575±0.339^a^ |
| C1 | 2-ethyl-Butanoic acid | - | 0 | 0 | 0 | 0 | 0 | 0 |
| C2 | 2-oxo-Propanoic acid | - | 4.642±0.487^a^ | 4.324±0.455^b^ | 3.981±0.421^b^ | 3.094±0.332^c^ | 2.84±0.307^c^ | 2.65±0.288^b^ |
| C3 | Pentanoic acid | 1733 | 0 | 0 | 0 | 0 | 0 | 0 |
| C4 | 2-methyl-Butanoic acid | - | 36.61±2.427^b^ | 36.402±2.712^b^ | 36.172±2.383^b^ | 44.234±3.189^b^ | 48.859±3.652^b^ | 99.8±8.746^a^ |
| C5 | Propanoic acid | 1535 | 0 | 0 | 0 | 0 | 0 | 0 |
| C6 | Heptanoic acid | 1950 | 11.601±0.4^b^ | 13.963±0.636^b^ | 11.187±0.983^d^ | 10.367±0.901^c^ | 9.367±0.801^b^ | 8.321±0.696^a^ |
| C7 | Nonanoic acid | 2171 | 16.429±1.546^b^ | 16.916±1.595^cd^ | 16.073±1.51^d^ | 59.765±5.88^a^ | 0 | 0 |
| C8 | 3-methyl-Butanoic acid | - | 0 | 32.999±2.924^c^ | 0 | 0 | 0 | 0 |
| C9 | Decanoic acid | 1097 | 47.664±3.094^b^ | 77.259±6.054^a^ | 57.057±4.034^c^ | 64.006±4.729^c^ | 105.801±8.908^a^ | 82.885±6.617^c^ |
| C10 | Butanoic acid | 1625 | 62.803±2.908^e^ | 87.912±5.419^d^ | 141.82±10.81^c^ | 178.753±14.503^c^ | 199.05±16.533^b^ | 186.646±15.293^c^ |
| C11 | Hexanoic acid | 1846 | 57.833±4.759^bc^ | 62.968±5.273^b^ | 75.911±6.567^b^ | 66.794±5.655^c^ | 82.729±7.249^ab^ | 96.205±8.597^a^ |
| C12 | Octanoic acid | 2060 | 8.306±0.307^f^ | 10.511±0.527^e^ | 11.913±0.667^e^ | 13.818±0.858^e^ | 10.94±0.57^e^ | 7.44±0.22^d^ |
| C13 | Acetic acid | 1449 | 66.834±5.659^e^ | 73.322±6.308^d^ | 79.473±6.923^d^ | 164.295±15.406^bc^ | 133.375±12.314^cd^ | 116.607±10.637^d^ |
| C14 | 2-methyl-Propanoic acid | - | 78.389±6.515^a^ | 85.197±7.196^a^ | 73.544±6.03^b^ | 74.628±6.139^a^ | 76.666±6.343^a^ | 87.197±7.396^a^ |
| D1 | 3-Heptanone | 1161 | 0 | 0 | 0 | 0 | 0 | 0 |
| D2 | dihydro-2-methyl-3(2H)-Furanone | - | 0.678±0.065^a^ | 1.483±0.135^a^ | 1.079±0.097^a^ | 0.973±0.094^a^ | 0.882±0.085^a^ | 0.792±0.076^a^ |
| D3 | 2-Octanone | 1287 | 0.76±0.072^b^ | 0.761±0.072^c^ | 0.764±0.066^c^ | 0.774±0.067^c^ | 0.819±0.072^b^ | 0 |
| D4 | 2-Pentanone | 981 | 0 | 0 | 0 | 0 | 0 | 0 |
| D5 | 2-Undecanone | 1598 | 13.14±0.877^b^ | 25.683±2.131^a^ | 24.382±2.001^ab^ | 23.451±1.908^b^ | 0 | 0 |
| D6 | 1-hydroxy-2-Propanone | - | 7.057±0.569^c^ | 5.388±0.402^bc^ | 1.329±0.142^de^ | 4.26±0.435^d^ | 5.7±0.579^c^ | 35.751±3.438^b^ |
| D7 | 2-Heptanone | 1182 | 116.403±7.249^b^ | 161.66±11.775^ab^ | 133.859±4.995^c^ | 120.086±3.618^d^ | 144.54±6.063^c^ | 209.968±11.616^c^ |
| D8 | 2-Nonanone | 1390 | 87.324±6.893^b^ | 105.317±8.141^a^ | 97.86±7.947^c^ | 100.292±7.638^b^ | 148.499±12.459^b^ | 157.736±13.383^b^ |
| D9 | 3-hydroxy-2-Butanone | - | 140.93±11.702^bc^ | 46.806±3.698^d^ | 47.436±3.761^c^ | 76.025±5.764^c^ | 102.661±7.875^d^ | 415.695±35.588^a^ |
| E1 | ACETIC ACID, METHYL ESTER | 828 | 0 | 0 | 0 | 0 | 0 | 0 |
| E2 | Pentanoic acid, ethyl ester | 1134 | 0 | 0 | 1.328±0.075^c^ | 7.692±0.711^a^ | 10.362158±0.053^a^ | 5.183±0.46^a^ |
| E3 | Butanoic acid, pentyl ester | 1305 | 0.445±0.036^b^ | 0.804±0.072^d^ | 1.097±0.052^d^ | 1.762±0.118^e^ | 0.891±0.025^e^ | 0.517±0.035^c^ |
| E4 | acetic acid, 2- methyl-propyl ester | 1012 | 0 | 7.698±0.712^b^ | 8.921±0.834^d^ | 9.198±0.862^c^ | 10.781±0.095^c^ | 11.628±0.18^c^ |
| E5 | 1,2-Benzenedicarboxylic acid-bis(2-methylpropyl) ester | 2536 | 11.236±0.141^b^ | 28.79±1.896^a^ | 8.247±0.767^d^ | 12.038±0.221^b^ | 15.378±0.555^a^ | 0 |
| E6 | Acetic acid, propyl ester | 973 | 64.257±4.587^a^ | 13.966±0.414^c^ | 3.007±0.243^c^ | 22.055±1.223^c^ | 0 | 0 |
| E7 | Butyrolactone | 1632 | 3.742±0.316^b^ | 6.015±0.544^c^ | 8.389±0.781^b^ | 10.363±0.653^b^ | 14.288±1.046^c^ | 17.956±1.413^c^ |
| E8 | 9-Decenoic acid, ethyl ester | 1694 | 27.645±2.382^d^ | 37.684±3.385^d^ | 67.387±5.5ｄ | 80.84±6.845^c^ | 0 | 0 |
| E9 | Nonanoic acid, ethyl ester | 1531 | 15.324±1.149^c^ | 18.806±1.498^c^ | 27.439±2.361^d^ | 32.714±2.888^b^ | 134.744±11.383^a^ | 0 |
| E10 | 2-hydroxy-Propanoic acid ethyl ester | 1347 | 55.764±4.337^c^ | 74.546±6.216^c^ | 112.561±9.165^b^ | 136.812±11.59^a^ | 290.41±25.66^b^ | 661.502±57.779^b^ |
| E11 | Butanoic acid, ethyl ester | 1035 | 192.273±17.136^d^ | 255.896±22.209^d^ | 276.974±24.316^d^ | 326.927±28.412^d^ | 325.367±28.256^d^ | 440.219±38.04^d^ |
| E12 | Heptanoic acid, ethyl ester | 1331 | 8.72±0.814^c^ | 16.429±1.26^c^ | 27.104±2.327^d^ | 34.484±3.065^c^ | 99.198±8.681^a^ | 66.457±5.407^d^ |
| E13 | Decanoic acid, ethyl ester | 1638 | 102.582±8.167^c^ | 122.594±10.168^d^ | 212.282±17.847^d^ | 268.897±23.509^c^ | 868.761±78.505^a^ | 350.124±30.731^b^ |
| E14 | Hexanoic acid, ethyl ester | 1233 | 95.153±8.276^d^ | 124.969±10.406^d^ | 169.563±14.865^d^ | 1713.853±152.104^a^ | 3405.495±314.869^a^ | 2643.504±238.669^c^ |
| E15 | 1-Butanol, 3-methyl-, acetate | 1122 | 213.639±17.983^d^ | 105.608±8.47^b^ | 140.704±11.979^d^ | 199.676±17.877^d^ | 187.887±16.698^c^ | 164.059±14.315^d^ |
| E16 | Acetic acid, butyl ester | 1074 | 76.093±6.37^d^ | 105.051±8.414^c^ | 87.419±7.503^d^ | 85.878±7.349^d^ | 87.269±7.488^bc^ | 118.669±9.776^b^ |
| E17 | Acetic acid, 2-phenylethyl ester | 1813 | 63.634±5.124^b^ | 81.954±6.956^c^ | 80.671±6.828^c^ | 97.364±8.497^b^ | 180.682±15.977^a^ | 141.438±12.053^a^ |
| E18 | Octanoic acid, ethyl ester | 1435 | 114.144±9.323^b^ | 145.943±12.503^c^ | 163.871±14.296^b^ | 151.128±13.022^bc^ | 137.465±11.656^c^ | 120.597±9.969^c^ |
| E19 | Acetic acid ethyl ester | 888 | 1076.678±92.276^a^ | 1103.066±94.915^a^ | 1003.12±84.92^a^ | 581.662±49.795^b^ | 563.93±48.022^b^ | 678.955±50.392^b^ |
| F1 | Benzene ethenyl | 1261 | 0 | 0 | 0 | 0 | 5.991±0.541^c^ | 0 |

|  |  |  | CSS | | | | | |
| --- | --- | --- | --- | --- | --- | --- | --- | --- |
|  | compound | RI | CSS-0 | CSS-10 | CSS-20 | CSS-30 | CSS-40 | CSS-50 |
| A1 | 2-Furanmethanol | 1660 | 0 | 0 | 0 | 0 | 0 | 0 |
| A2 | 1-nonanol | 1660 | 0 | 0 | 0 | 10.957±1.081^b^ | 0 | 0 |
| A3 | 1-Octanol | 1557 | 0.593±0.021^c^ | 1.082±0.129^c^ | 1.298±0.112^b^ | 0.987±0.092^c^ | 0.867±0.067^a^ | 0.772±0.062^a^ |
| A4 | 2,3-Butanediol | 1543 | 0.578±0.067^a^ | 0.581±0.067^a^ | 0.562±0.065^a^ | 0.516±0.061^a^ | 0.672±0.076^c^ | 0.687±0.078^b^ |
| A5 | 3-methyl-1-Pentanol | - | 3.426±0.328^c^ | 4.018±0.387^c^ | 4.198±0.443^c^ | 4.625±0.486^b^ | 2.198±0.243^c^ | 0 |
| A6 | 1-Pentanol | 1250 | 14.572±1.554^a^ | 23.893±2.486^a^ | 23.673±2.464^a^ | 23.474±2.444^a^ | 23.84±2.481^a^ | 17.513±1.848^a^ |
| A7 | 2-Pentanol | 1119 | 0.603±0.064^d^ | 1.77±0.149^c^ | 2.375±0.21^d^ | 2.741±0.246^bc^ | 1.087±0.081^c^ | 0.789±0.083^b^ |
| A8 | 1-Hexanol | 1355 | 0 | 0.231±0.026^b^ | 0.652±0.036^b^ | 1.231±0.144^b^ | 1.449±0.166^c^ | 1.227±0.144^c^ |
| A9 | 3-(methylthio)-1-Propanol | 1719 | 0 | 8.835±0.847^a^ | 8.936±0.857^a^ | 9.668±0.93^a^ | 12.353±1.198^a^ | 13.933±1.356^a^ |
| A10 | 1-Butanol | 1142 | 2.512±0.287^b^ | 1.658±0.202^b^ | 1.673±0.203^b^ | 1.531±0.189^c^ | 5.836±0.62^a^ | 6.946±0.731^a^ |
| A11 | 2-Nonanol | 1521 | 4.145±0.447^d^ | 8.423±0.874^c^ | 19.995±2.032^a^ | 12.894±1.321^b^ | 3.08±0.34^d^ | 2.098±0.242^d^ |
| A12 | 1,2-Propanediol | 1600 | 5.411±0.568^a^ | 2.073±0.234^c^ | 18.289±1.856^a^ | 38.29±3.856^a^ | 49.247±3.898^a^ | 91.379±8.111^ab^ |
| A13 | 2-methyl-1-Propanol | - | 85.502±5.577^c^ | 85.079±5.535^c^ | 96.236±6.651^c^ | 81.967±5.224^c^ | 81.854±5.212^c^ | 87.413±5.768^c^ |
| A14 | 2-Heptanol | 1320 | 10.468±1.078^b^ | 15.378±1.569^b^ | 19.288±1.96^c^ | 22.604±2.291^b^ | 10.048±1.036^c^ | 12.658±1.297^c^ |
| A15 | 3-methyl- 2-Butanol | - | 2.299±0.187^e^ | 8.936±0.851^a^ | 7.903±0.747^b^ | 1.099±0.067^e^ | 7.477±0.705^a^ | 16.219±1.579^a^ |
| A16 | Benzeneethanol | 1906 | 149.023±12.516^d^ | 296.023±27.216^b^ | 276.928±25.307^b^ | 309.074±28.521^b^ | 435.158±41.13^ab^ | 505.74±48.188^a^ |
| A17 | ISO AMYL ALCOHOL | 1209 | 1675.09±123.527^a^ | 1658.797±121.898^ab^ | 2137.553±169.773^a^ | 1763.289±132.347^a^ | 3230.288±279.047^a^ | 3193.378±275.356^a^ |
| B1 | 2-furan-carboxaldehyde | 1461 | 0 | 0 | 0 | 0 | 0 | 0 |
| B2 | 2-Hexenal | 1090 | 0 | 0 | 0 | 0 | 0 | 0 |
| B3 | Octanal | 1289 | 0 | 0 | 0 | 0 | 0 | 0 |
| B4 | Hexanal | 1083 | 0 | 0 | 3.891±0.316^b^ | 5.374±0.464^b^ | 8.375±0.765^a^ | 12.352±0.562^b^ |
| B5 | Nonanal | 1391 | 13.34±0.842^c^ | 16.815±1.19^a^ | 12.284±0.736^a^ | 8.136±0.776^c^ | 32.985±3.261^a^ | 32.031±3.165^a^ |
| B6 | Acetaldehyde | 702 | 1.113±0.092^d^ | 1.433±0.124^b^ | 3.177±0.299^a^ | 1.782±0.159^c^ | 1.207±0.102^c^ | 2.18±0.199^b^ |
| C1 | 2-ethyl-Butanoic acid | - | 0 | 0 | 0 | 0 | 0 | 0 |
| C2 | 2-oxo-Propanoic acid | - | 2.152±0.238^c^ | 2.981±0.321^c^ | 3.233±0.346^c^ | 3.982±0.421^b^ | 4.098±0.433^b^ | 4.125±0.436^a^ |
| C3 | Pentanoic acid | 1733 | 0 | 0 | 0 | 0 | 0 | 0 |
| C4 | 2-methyl-Butanoic acid | - | 0 | 0.834±0.083^d^ | 0.918±0.092^d^ | 1.097±0.11^d^ | 1.219±0.122^d^ | 1.314±0.131^d^ |
| C5 | Propanoic acid | 1535 | 13.332±1.294^a^ | 16.254±1.586^a^ | 16.275±1.589^a^ | 15.156±1.477^a^ | 14.027±1.364^a^ | 13.927±1.354^a^ |
| C6 | Heptanoic acid | 1950 | 0 | 24.62±1.702^a^ | 20.649±1.929^a^ | 0 | 0 | 0 |
| C7 | Nonanoic acid | 2171 | 11.87±1.09^b^ | 67.43±6.646^a^ | 55.152±5.418^a^ | 15.052±1.408^d^ | 17.446±1.648^b^ | 0 |
| C8 | 3-methyl-Butanoic acid | - | 23.853±2.009^c^ | 44.028±4.027^b^ | 31.505±2.775^b^ | 33.046±2.929^ab^ | 62.598±5.884^a^ | 71.917±6.816^b^ |
| C9 | Decanoic acid | 1097 | 39.296±2.258^c^ | 64.021±4.73^bc^ | 68.378±5.166^b^ | 70.552±5.383^bc^ | 104.536±8.782^a^ | 102.864±8.614^b^ |
| C10 | Butanoic acid | 1625 | 112.782±7.906^d^ | 183.488±14.977^b^ | 208.625±17.491^b^ | 224.709±19.099^b^ | 269.306±23.559^a^ | 261.646±22.793^a^ |
| C11 | Hexanoic acid | 1846 | 93.701±8.346^a^ | 95.828±8.559^a^ | 109.63±9.939^b^ | 107.349±9.711^a^ | 88.614±7.837^a^ | 76.732±6.649^b^ |
| C12 | Octanoic acid | 2060 | 12.155±0.692^e^ | 21.314±1.607^c^ | 21.367±1.613^d^ | 22.515±1.728^d^ | 26.037±2.08^d^ | 33.989±2.875^b^ |
| C13 | Acetic acid | 1449 | 148.407±13.817^c^ | 170.84±16.06^b^ | 181.981±17.174^b^ | 239.86±22.962^a^ | 195.341±18.51^b^ | 190.229±17.999^b^ |
| C14 | 2-methyl-Propanoic acid | - | 32.46±1.922^c^ | 78.467±6.523^a^ | 52.048±3.881^c^ | 64.325±5.109^b^ | 63.667±5.043^b^ | 51.137±3.79^c^ |
| D1 | 3-Heptanone | 1161 | 0 | 0 | 0 | 0 | 0 | 0 |
| D2 | dihydro-2-methyl-3(2H)-Furanone | - | 0 | 0 | 0 | 0 | 0 | 0 |
| D3 | 2-Octanone | 1287 | 0.908±0.087^b^ | 1.054±0.101^b^ | 1.287±0.119^b^ | 1.374±0.127^ab^ | 1.416±0.132^b^ | 1.425±0.133^b^ |
| D4 | 2-Pentanone | 981 | 22.046±1.681^b^ | 23.615±1.838^b^ | 17.568±1.233^b^ | 16.263±1.102^b^ | 5.288±0.44^bc^ | 3.481±0.259^b^ |
| D5 | 2-Undecanone | 1598 | 11.708±0.734^b^ | 20.962±1.659^c^ | 27.624±2.325^a^ | 26.63±2.226^ab^ | 0 | 0 |
| D6 | 1-hydroxy-2-Propanone | - | 10.465±0.91^a^ | 17.743±1.637^a^ | 11.283±0.991^a^ | 20.033±1.866^a^ | 26.332±2.496^a^ | 29.398±2.803^c^ |
| D7 | 2-Heptanone | 1182 | 157.11±11.32^a^ | 166.856±12.295^a^ | 259.081±16.527^a^ | 236.867±14.306^a^ | 317.958±23.515^a^ | 377.765±29.496^a^ |
| D8 | 2-Nonanone | 1390 | 82.837±6.445^b^ | 104.542±8.063^a^ | 129.553±10.564^a^ | 130.125±10.622^a^ | 186.288±16.238^a^ | 216.898±18.309^a^ |
| D9 | 3-hydroxy-2-Butanone | - | 119.66±9.575^c^ | 167.32±14.341^b^ | 101.896±7.799^b^ | 150.752±12.684^b^ | 202.915±16.911^c^ | 425.639±36.582^a^ |
| E1 | ACETIC ACID, METHYL ESTER | 828 | 0 | 0 | 0 | 0 | 0 | 0 |
| E2 | Pentanoic acid, ethyl ester | 1134 | 3.274±0.269^a^ | 2.917±0.234^a^ | 2.071±0.149^a^ | 1.832±0.125^b^ | 1.092±0.051^d^ | 0.894±0.031^d^ |
| E3 | Butanoic acid, pentyl ester | 1305 | 0 | 0 | 8.31±0.773^c^ | 18.375±0.855^b^ | 23.375±1.355^c^ | 39.208±2.938^a^ |
| E4 | acetic acid, 2- methyl-propyl ester | 1012 | 36.517±2.669^b^ | 38.152±2.832^a^ | 40.125±3.03^a^ | 36.182±2.635^b^ | 32.892±2.306^b^ | 30.891±2.106^b^ |
| E5 | 1,2-Benzenedicarboxylic acid-bis(2-methylpropyl) ester | 2536 | 0 | 13.221±0.339^c^ | 17.684±0.785^a^ | 17.381±0.755^a^ | 0 | 0 |
| E6 | Acetic acid, propyl ester | 973 | 0 | 0 | 0 | 0 | 0 | 0 |
| E7 | Butyrolactone | 1632 | 0 | 14.106±1.028^a^ | 7.343±0.676^bc^ | 14.23±1.04^a^ | 13.39±0.956^c^ | 12.832±0.9^d^ |
| E8 | 9-Decenoic acid, ethyl ester | 1694 | 42.031±3.82^c^ | 57.003±4.461^c^ | 85.87±7.348^c^ | 89.698±7.731^c^ | 0 | 0 |
| E9 | Nonanoic acid, ethyl ester | 1531 | 0 | 0 | 34.437±3.061^c^ | 0 | 0 | 0 |
| E10 | 2-hydroxy-Propanoic acid ethyl ester | 1347 | 68.364±5.597^b^ | 83.769±7.138^bc^ | 90.299±7.791^c^ | 119.41±9.85^a^ | 261.901±22.809^b^ | 383.932±34.112^d^ |
| E11 | Butanoic acid, ethyl ester | 1035 | 213.718±17.991^d^ | 248.613±21.48^d^ | 274.695±24.089^d^ | 219.119±18.531^d^ | 216.781±18.297^d^ | 206.004±17.219^e^ |
| E12 | Heptanoic acid, ethyl ester | 1331 | 22.977±1.915^b^ | 30.013±2.618^b^ | 35.816±3.199^c^ | 38.836±3.501^c^ | 67.259±5.487^b^ | 90.282±7.789^bc^ |
| E13 | Decanoic acid, ethyl ester | 1638 | 111.929±9.102^c^ | 134.087±11.318^d^ | 201.108±16.73^d^ | 225.768±19.196^c^ | 346.816±30.401^c^ | 442.202±38.238^b^ |
| E14 | Hexanoic acid, ethyl ester | 1233 | 480.687±42.087^c^ | 608.847±52.514^c^ | 865.06±78.135^c^ | 1058.125±90.421^d^ | 1624.601±143.179^c^ | 2240.251±198.344^cd^ |
| E15 | 1-Butanol, 3-methyl-, acetate | 1122 | 493.778±43.396^b^ | 452.459±39.264^a^ | 489.08±42.926^ab^ | 963.13±87.942^a^ | 1631.756±143.895^a^ | 1722.737±152.993^a^ |
| E16 | Acetic acid, butyl ester | 1074 | 195.49±17.458^a^ | 172.115±15.121^a^ | 240.913±20.71^a^ | 213.02±17.921^a^ | 196.84±17.593^a^ | 232.874±19.906^a^ |
| E17 | Acetic acid, 2-phenylethyl ester | 1813 | 8.714±0.813^c^ | 14.049±1.022^d^ | 13.679±0.985^d^ | 13.314±0.948^d^ | 24.313±2.048^d^ | 18.957±1.513^e^ |
| E18 | Octanoic acid, ethyl ester | 1435 | 95.183±8.279^b^ | 116.808±9.59^d^ | 179.707±15.88^ab^ | 189.12±16.821^a^ | 179.667±15.876^ab^ | 159.392±13.848^b^ |
| E19 | Acetic acid ethyl ester | 888 | 526.642±44.293^bc^ | 711.153±62.744^b^ | 772.426±68.872^b^ | 663.564±57.985^b^ | 481.556±42.174^b^ | 211.887±20.381^e^ |
| F1 | Benzene ethenyl | 1261 | 5.157±0.458^c^ | 7.895±0.732^d^ | 9.189±0.861^c^ | 8.188±0.761^b^ | 7.954±0.737^c^ | 6.202±0.562^d^ |

|  |  |  | CSM:CSS 1:1 | | | | | |
| --- | --- | --- | --- | --- | --- | --- | --- | --- |
|  | compound | RI | CSM:CSS 1:1-0 | CSM:CSS 1:1-10 | CSM:CSS 1:1-20 | CSM:CSS 1:1-30 | CSM:CSS 1:1-40 | CSM:CSS 1:1-50 |
| A1 | 2-Furanmethanol | 1660 | 0 | 0 | 0 | 0 | 0 | 0 |
| A2 | 1-nonanol | 1660 | 0 | 0 | 0 | 0 | 32.777±3.271^a^ | 37.123±3.211^a^ |
| A3 | 1-Octanol | 1557 | 3.147±0.361^b^ | 5.275±0.532^a^ | 0 | 0 | 0 | 0 |
| A4 | 2,3-Butanediol | 1543 | 0 | 0 | 0 | 0 | 2.881±0.327^a^ | 0 |
| A5 | 3-methyl-1-Pentanol | - | 4.178±0.403^b^ | 7.918±0.777^a^ | 7.681±0.791^a^ | 7.309±0.754^a^ | 7.192±0.742^a^ | 0 |
| A6 | 1-Pentanol | 1250 | 0 | 0 | 0 | 0 | 0 | 0 |
| A7 | 2-Pentanol | 1119 | 1.784±0.15^c^ | 4.264±0.398^b^ | 7.6±0.732^a^ | 3.192±0.291^b^ | 2.193±0.191^b^ | 0 |
| A8 | 1-Hexanol | 1355 | 0 | 0 | 0 | 0 | 38.533±3.583^a^ | 57.202±5.45^a^ |
| A9 | 3-(methylthio)-1-Propanol | 1719 | 4.165±0.38^a^ | 4.697±0.433^c^ | 5.832±0.546^bc^ | 6.963±0.659^b^ | 7.418±0.705^c^ | 12.096±1.073^ab^ |
| A10 | 1-Butanol | 1142 | 2.305±0.267^b^ | 2.588±0.295^a^ | 2.918±0.328^a^ | 4.091±0.445^a^ | 5.083±0.544^ab^ | 6.092±0.645^a^ |
| A11 | 2-Nonanol | 1521 | 16.452±1.677^b^ | 18.865±1.919^a^ | 21.572±2.189^a^ | 22.462±2.278^a^ | 23.959±2.428^b^ | 25.094±2.541^a^ |
| A12 | 1,2-Propanediol | 1600 | 3.651±0.392^b^ | 5.289±0.556^a^ | 8.379±0.865^b^ | 10.289±1.056^b^ | 12.062±1.233^c^ | 80.177±6.991^bc^ |
| A13 | 2-methyl-1-Propanol | - | 130.019±10.029^a^ | 140.189±11.046^b^ | 161.561±13.183^a^ | 185.576±15.585^a^ | 195.378±16.565^a^ | 230.129±20.04^b^ |
| A14 | 2-Heptanol | 1320 | 18.59±1.89^a^ | 22.979±2.329^a^ | 28.806±2.912^a^ | 31.805±3.212^a^ | 38.176±3.849^a^ | 45.618±4.593^b^ |
| A15 | 3-methyl- 2-Butanol | - | 19.717±1.929^a^ | 7.726±0.73^a^ | 7.507±0.708^b^ | 11.002±1.057^b^ | 6.865±0.644^a^ | 5.814±0.538^b^ |
| A16 | Benzeneethanol | 1906 | 258.879±23.502^b^ | 293.077±26.922^b^ | 285.467±26.161^b^ | 305.34±28.148^b^ | 399.674±37.581^b^ | 361.351±33.749^b^ |
| A17 | ISO AMYL ALCOHOL | 1209 | 1455.057±101.524^b^ | 1774.227±133.441^a^ | 1722.047±128.223^b^ | 1517.71±107.789^b^ | 2987.676±254.786^a^ | 2954.09±251.427^a^ |
| B1 | 2-furan-carboxaldehyde | 1461 | 0 | 0 | 0 | 0 | 0 | 0 |
| B2 | 2-Hexenal | 1090 | 0 | 0 | 0 | 0 | 0 | 0 |
| B3 | Octanal | 1289 | 0 | 0 | 0 | 0 | 10.685±1.001^a^ | 0 |
| B4 | Hexanal | 1083 | 0 | 0 | 0 | 0 | 0 | 0 |
| B5 | Nonanal | 1391 | 0 | 10.5±0.558^c^ | 12.216±0.73^a^ | 12.09±1.171^a^ | 16.58±1.62^c^ | 17.902±1.752^c^ |
| B6 | Acetaldehyde | 702 | 2.937±0.275^c^ | 1.279±0.109^b^ | 1.359±0.117^c^ | 4.356±0.417^a^ | 3.739±0.355^a^ | 0 |
| C1 | 2-ethyl-Butanoic acid | - | 0 | 0 | 0 | 0 | 0 | 0 |
| C2 | 2-oxo-Propanoic acid | - | 5.229±0.546^a^ | 5.125±0.536^a^ | 5.012±0.524^a^ | 4.94±0.517^a^ | 4.877±0.511^a^ | 4.579±0.481^a^ |
| C3 | Pentanoic acid | 1733 | 0 | 0 | 0 | 0 | 0 | 0 |
| C4 | 2-methyl-Butanoic acid | - | 43.263±3.092^a^ | 49.862±4.058^a^ | 57.831±4.855^a^ | 67.981±5.87^a^ | 72.628±6.335^a^ | 79.614±7.033^b^ |
| C5 | Propanoic acid | 1535 | 0 | 0 | 0 | 0 | 0 | 0 |
| C6 | Heptanoic acid | 1950 | 25.78±1.818^a^ | 23.841±1.624^a^ | 17.432±1.607^b^ | 12.999±0.54^b^ | 10.278±0.892^b^ | 0 |
| C7 | Nonanoic acid | 2171 | 48.945±4.798^a^ | 59.511±5.854^b^ | 30.279±2.931^b^ | 38.043±3.707^b^ | 40.278±3.931^a^ | 45.893±4.492^a^ |
| C8 | 3-methyl-Butanoic acid | - | 27.759±2.4^bc^ | 35.057±3.13^c^ | 48.684±4.492^a^ | 35.68±3.192^a^ | 32.389±2.863^b^ | 37.673±3.391^c^ |
| C9 | Decanoic acid | 1097 | 47.682±3.096^b^ | 57.586±4.087^c^ | 74.616±5.79^ab^ | 82.567±6.585^a^ | 105.933±8.921^a^ | 177.149±16.043^a^ |
| C10 | Butanoic acid | 1625 | 263.946±23.023^a^ | 292.239±25.852^a^ | 300.452±26.673^a^ | 338.759±30.504^a^ | 268.818±23.51^a^ | 244.342±21.062^ab^ |
| C11 | Hexanoic acid | 1846 | 82.556±7.232^a^ | 89.45±7.921^a^ | 94.681±8.444^b^ | 91.166±8.093^b^ | 85.154±7.491^ab^ | 76.335±6.61^b^ |
| C12 | Octanoic acid | 2060 | 39.296±3.406^a^ | 43.368±3.813^a^ | 45.141±3.99^a^ | 46.894±4.165^a^ | 48.64±4.34^a^ | 49.726±4.449^a^ |
| C13 | Acetic acid | 1449 | 186.606±17.637^b^ | 199.754±18.951^ab^ | 207.569±19.733^ab^ | 190.445±18.021^b^ | 175.008±16.477^b^ | 149.877±13.964^c^ |
| C14 | 2-methyl-Propanoic acid | - | 47.938±3.47^b^ | 54.336±4.11^c^ | 83.862±7.062^a^ | 61.22±4.798^b^ | 84.446±7.121^a^ | 89.377±7.614^a^ |
| D1 | 3-Heptanone | 1161 | 0 | 0 | 0 | 0 | 0 | 0 |
| D2 | dihydro-2-methyl-3(2H)-Furanone | - | 0 | 0 | 0 | 0 | 0 | 0 |
| D3 | 2-Octanone | 1287 | 1.098±0.106^a^ | 1.293±0.125^a^ | 1.483±0.138^a^ | 1.569±0.147^a^ | 1.672±0.157^b^ | 1.792±0.169^a^ |
| D4 | 2-Pentanone | 981 | 16.782±1.154^b^ | 12.891±0.765^c^ | 10.982±0.574^c^ | 8.082±0.284^c^ | 6.897±0.601^b^ | 5.824±0.493^a^ |
| D5 | 2-Undecanone | 1598 | 22.523±1.815^a^ | 25.279±2.091^ab^ | 27.551±2.318^a^ | 29.892±2.552^a^ | 30.289±2.592^a^ | 0 |
| D6 | 1-hydroxy-2-Propanone | - | 3.809±0.244^e^ | 5.378±0.401^bc^ | 9.297±0.793^b^ | 12.13±1.076^c^ | 12.906±1.154^b^ | 43.121±4.175^a^ |
| D7 | 2-Heptanone | 1182 | 128.57±8.466^b^ | 166.471±12.256^a^ | 134.624±5.071^c^ | 166.012±8.21^b^ | 238.098±14.429^b^ | 257.762±16.395^b^ |
| D8 | 2-Nonanone | 1390 | 83.32±6.493^b^ | 109.736±8.583^a^ | 115.837±9.193^ab^ | 120.127±9.622^a^ | 161.537±13.763^b^ | 173.238±14.933^b^ |
| D9 | 3-hydroxy-2-Butanone | - | 78.748±6.036^d^ | 75.8±5.741^c^ | 101.027±7.712^b^ | 111.032±8.712^c^ | 186.242±16.233^c^ | 225.803±19.199^c^ |
| E1 | ACETIC ACID, METHYL ESTER | 828 | 0 | 0 | 0 | 0 | 0 | 0 |
| E2 | Pentanoic acid, ethyl ester | 1134 | 1.398±0.082^b^ | 1.438±0.086^b^ | 1.572±0.099^b^ | 1.604±0.102^b^ | 1.504±0.092^c^ | 1.321±0.074^bc^ |
| E3 | Butanoic acid, pentyl ester | 1305 | 20.318±1.049^a^ | 25.416±1.559^a^ | 28.092±1.826^a^ | 34.193±2.436^a^ | 38.294±2.846^a^ | 40.473±3.064^a^ |
| E4 | acetic acid, 2- methyl-propyl ester | 1012 | 52.692±3.43^a^ | 41.037±3.121^a^ | 33.079±2.325^b^ | 45.203±3.537^a^ | 58.412±4.002^a^ | 76.991±5.86^a^ |
| E5 | 1,2-Benzenedicarboxylic acid-bis(2-methylpropyl) ester | 2536 | 15.435±0.561^a^ | 21.654±1.182^b^ | 10.222±0.039^c^ | 8.59±0.801^c^ | 6.892±0.631^b^ | 0 |
| E6 | Acetic acid, propyl ester | 973 | 38.123±2.829^b^ | 42.754±3.292^b^ | 64.31±4.592^a^ | 70.91±5.252^a^ | 78.671±6.028^a^ | 80.092±6.17^a^ |
| E7 | Butyrolactone | 1632 | 0 | 4.261±0.368^d^ | 6.57±0.599^c^ | 10.354±0.652^b^ | 14.836±1.101^c^ | 19.082±1.525^bc^ |
| E8 | 9-Decenoic acid, ethyl ester | 1694 | 154.112±13.32^a^ | 188.2±16.729^a^ | 159.48±13.857^a^ | 175.093±15.418^a^ | 220.754±18.694^a^ | 0 |
| E9 | Nonanoic acid, ethyl ester | 1531 | 23.216±1.939^b^ | 54.08±4.169^a^ | 57.839±4.545^a^ | 63.519±5.113^a^ | 72.057±5.967^b^ | 93.314±8.092^a^ |
| E10 | 2-hydroxy-Propanoic acid ethyl ester | 1347 | 84.169±7.178^a^ | 94.256±8.187^b^ | 79.346±6.696^c^ | 119.658±9.875^a^ | 113.032±9.212^c^ | 172.997±15.209^e^ |
| E11 | Butanoic acid, ethyl ester | 1035 | 364.579±32.177^c^ | 495.86±43.604^c^ | 512.338±42.863^c^ | 687.325±60.362^c^ | 706.876±62.317^c^ | 795.134±71.142^c^ |
| E12 | Heptanoic acid, ethyl ester | 1331 | 23.195±1.937^b^ | 44.809±4.098^a^ | 45.19±4.136^b^ | 47.871±4.404^b^ | 93.069±8.068^a^ | 101.565±8.066^b^ |
| E13 | Decanoic acid, ethyl ester | 1638 | 228.436±19.463^a^ | 392.793±34.998^a^ | 375.834±33.302^a^ | 477.047±41.723^a^ | 666.041±58.233^b^ | 710.344±62.663^a^ |
| E14 | Hexanoic acid, ethyl ester | 1233 | 1206.441±105.252^a^ | 1330.143±117.622^a^ | 1260.82±110.69^b^ | 1404.646±125.073^c^ | 1601.233±140.842^c^ | 1846.475±165.367^d^ |
| E15 | 1-Butanol, 3-methyl-, acetate | 1122 | 332.108±28.93^c^ | 460.526±40.071^a^ | 450.873±39.105^bc^ | 425.301±36.548^c^ | 697.604±61.389^b^ | 742.288±65.858^c^ |
| E16 | Acetic acid, butyl ester | 1074 | 133.302±11.239^b^ | 114.152±9.324^c^ | 128.279±10.737^c^ | 144.735±12.383^b^ | 72.144±5.975^cd^ | 88.469±7.608^c^ |
| E17 | Acetic acid, 2-phenylethyl ester | 1813 | 60.33±4.794^b^ | 78.082±6.569^c^ | 125.333±10.442^a^ | 83.119±7.073^c^ | 194.492±17.358^a^ | 122.354±10.144^b^ |
| E18 | Octanoic acid, ethyl ester | 1435 | 159.726±13.882^a^ | 179.481±15.857^ab^ | 198.002±17.709^a^ | 191.237±17.033^a^ | 185.699±16.479^a^ | 182.664±16.175^a^ |
| E19 | Acetic acid ethyl ester | 888 | 583.787±50.008^b^ | 601.446±51.774^c^ | 657.997±57.429^c^ | 759.089±67.538^a^ | 845.684±76.197^a^ | 933.04±68.383^a^ |
| F1 | Benzene ethenyl | 1261 | 26.035±2.221^b^ | 42.492±3.866^c^ | 48.804±4.497^b^ | 49.164±4.533^a^ | 67.289±5.49^a^ | 70.109±5.772^b^ |

|  |  |  | CSM:CSS 1:2 | | | | | |
| --- | --- | --- | --- | --- | --- | --- | --- | --- |
|  | compound | RI | CSM:CSS 1:2-0 | CSM:CSS 1:2-10 | CSM:CSS 1:2-20 | CSM:CSS 1:2-30 | CSM:CSS 1:2-40 | CSM:CSS 1:2-50 |
| A1 | 2-Furanmethanol | 1660 | 0 | 0 | 0 | 0 | 0 | 0 |
| A2 | 1-nonanol | 1660 | 0 | 0 | 16.139±1.382^a^ | 0 | 0 | 0 |
| A3 | 1-Octanol | 1557 | 0 | 0 | 0 | 8.031±0.738^a^ | 0 | 0 |
| A4 | 2,3-Butanediol | 1543 | 0 | 0 | 0 | 0 | 0 | 66.815±6.972^a^ |
| A5 | 3-methyl-1-Pentanol | - | 0 | 6.654±0.65^b^ | 6.108±0.634^b^ | 6.089±0.632^a^ | 5.927±0.616^b^ | 0 |
| A6 | 1-Pentanol | 1250 | 0 | 0 | 0 | 0.651±0.072^b^ | 13.278±1.425^b^ | 18.604±1.957^a^ |
| A7 | 2-Pentanol | 1119 | 5.57±0.529^a^ | 4.982±0.47^ab^ | 3.986±0.371^c^ | 2.209±0.193^cd^ | 1.926±0.165^b^ | 0 |
| A8 | 1-Hexanol | 1355 | 3.588±0.38^a^ | 5.378±0.559^a^ | 14.378±1.459^a^ | 22.822±2.012^a^ | 33.284±3.058^b^ | 44.684±4.198^b^ |
| A9 | 3-(methylthio)-1-Propanol | 1719 | 0 | 7.328±0.696^b^ | 5.169±0.48^c^ | 6.831±0.646^b^ | 7.518±0.715^c^ | 11.62±1.125^ab^ |
| A10 | 1-Butanol | 1142 | 2.153±0.251^b^ | 2.678±0.304^a^ | 2.981±0.334^a^ | 3.71±0.407^ab^ | 4.388±0.475^b^ | 0 |
| A11 | 2-Nonanol | 1521 | 0 | 14.843±1.516^b^ | 10.278±1.06^bc^ | 5.982±0.63^c^ | 3.75±0.407^d^ | 0 |
| A12 | 1,2-Propanediol | 1600 | 4.709±0.498^a^ | 4.672±0.494^ab^ | 4.619±0.489^c^ | 6.3±0.657^c^ | 8.478±0.875^d^ | 96.311±8.604^a^ |
| A13 | 2-methyl-1-Propanol | - | 68.18±3.845^d^ | 98.551±6.882^c^ | 91.975±6.225^c^ | 69.552±3.982^c^ | 0 | 0 |
| A14 | 2-Heptanol | 1320 | 8.805±0.912^b^ | 15.389±1.57^b^ | 19.289±1.96^c^ | 22.107±2.242^b^ | 24.106±2.442^b^ | 39.627±3.994^b^ |
| A15 | 3-methyl- 2-Butanol | - | 15.024±1.459^b^ | 4.579±0.415^b^ | 4.748±0.432^c^ | 3.579±0.315^d^ | 3.127±0.27^c^ | 3.036±0.261^c^ |
| A16 | Benzeneethanol | 1906 | 216.304±19.244^c^ | 366.714±34.285^a^ | 350.468±32.661^a^ | 373.092±34.923^a^ | 329.569±30.571^c^ | 313.625±28.977^b^ |
| A17 | ISO AMYL ALCOHOL | 1209 | 1438.509±99.869^b^ | 1477.217±103.74^b^ | 1684.336±124.452^b^ | 1243.488±80.367^c^ | 1142.627±70.281^c^ | 1099.863±66.004^b^ |
| B1 | 2-furan-carboxaldehyde | 1461 | 0 | 0 | 0 | 0 | 0 | 0 |
| B2 | 2-Hexenal | 1090 | 0 | 0 | 0 | 0 | 0 | 0 |
| B3 | Octanal | 1289 | 0 | 0 | 4.515±0.47^a^ | 0 | 0 | 9.077±0.84^a^ |
| B4 | Hexanal | 1083 | 0 | 0 | 0 | 0 | 0 | 4.626±0.39^c^ |
| B5 | Nonanal | 1391 | 0 | 14.03±0.911^b^ | 0.57±0.005^e^ | 7.019±0.664^c^ | 15.666±1.529^c^ | 25.659±2.528^b^ |
| B6 | Acetaldehyde | 702 | 5.371±0.518^a^ | 1.267±0.108^b^ | 1.069±0.088^d^ | 1.633±0.144^c^ | 2.843±0.265^b^ | 3.725±0.354^a^ |
| C1 | 2-ethyl-Butanoic acid | - | 0 | 0 | 0 | 0 | 0 | 0 |
| C2 | 2-oxo-Propanoic acid | - | 3.671±0.39^b^ | 3.293±0.352^c^ | 3.019±0.325^c^ | 2.981±0.321^c^ | 2.381±0.261^c^ | 2.198±0.243^b^ |
| C3 | Pentanoic acid | 1733 | 0 | 0 | 0 | 0 | 0 | 0 |
| C4 | 2-methyl-Butanoic acid | - | 20.125±1.779^c^ | 28.671±1.939^c^ | 29.891±2.061^c^ | 30.183±2.09^c^ | 31.291±2.201^c^ | 32.873±2.359^c^ |
| C5 | Propanoic acid | 1535 | 0 | 0 | 0 | 0 | 0 | 0 |
| C6 | Heptanoic acid | 1950 | 0 | 0 | 14.902±1.354^c^ | 0 | 0 | 0 |
| C7 | Nonanoic acid | 2171 | 15.269±1.43^b^ | 20.702±1.973^c^ | 24.565±2.36^c^ | 29.801±2.883^c^ | 0 | 0 |
| C8 | 3-methyl-Butanoic acid | - | 32.193±2.843^b^ | 38.396±3.464^bc^ | 46.773±4.301^a^ | 28.226±2.447^b^ | 22.877±1.912^c^ | 90.156±8.64^a^ |
| C9 | Decanoic acid | 1097 | 41.518±2.48^bc^ | 67.499±5.078^b^ | 69.437±5.272^ab^ | 81.414±6.469^a^ | 36.707±1.999^b^ | 108.778±9.206^b^ |
| C10 | Butanoic acid | 1625 | 138.271±10.455^c^ | 155.657±12.194^bc^ | 185.227±15.151^b^ | 160.668±12.695^c^ | 152.83±11.911^c^ | 142.431±10.871^d^ |
| C11 | Hexanoic acid | 1846 | 69.359±5.912^b^ | 90.314±8.007^a^ | 93.825±8.359^b^ | 86.782±7.654^b^ | 79.668±6.943^ab^ | 65.979±5.574^b^ |
| C12 | Octanoic acid | 2060 | 22.402±1.716^c^ | 24.066±1.883^c^ | 27.487±2.225^c^ | 29.505±2.427^c^ | 31.419±2.618^c^ | 35.129±2.989^b^ |
| C13 | Acetic acid | 1449 | 165.525±15.529^bc^ | 177.982±16.774^ab^ | 183.716±17.348^b^ | 175.819±16.558^b^ | 163.15±15.291^bc^ | 132.454±12.221^cd^ |
| C14 | 2-methyl-Propanoic acid | - | 55.577±4.234^b^ | 64.302±5.106^b^ | 71.693±5.845^b^ | 48.83±3.559^c^ | 32.682±1.944^c^ | 45.614±3.237^c^ |
| D1 | 3-Heptanone | 1161 | 0 | 0 | 0 | 0 | 0 | 0 |
| D2 | dihydro-2-methyl-3(2H)-Furanone | - | 0 | 0 | 0 | 0 | 0 | 0 |
| D3 | 2-Octanone | 1287 | 0 | 1.092±0.105^b^ | 1.119±0.102^b^ | 1.219±0.112^b^ | 1.413±0.131^b^ | 1.526±0.143^ab^ |
| D4 | 2-Pentanone | 981 | 0 | 0 | 0 | 0 | 4.22±0.333^c^ | 0 |
| D5 | 2-Undecanone | 1598 | 12.162±0.779^b^ | 24.797±2.043^ab^ | 23.259±1.889^b^ | 29.757±2.539^a^ | 0 | 0 |
| D6 | 1-hydroxy-2-Propanone | - | 5.016±0.365^d^ | 4.519±0.315^c^ | 8.102±0.673^c^ | 22.261±2.089^a^ | 3.777±0.387^c^ | 34.875±3.351^bc^ |
| D7 | 2-Heptanone | 1182 | 132.383±8.847^b^ | 144.463±10.055^b^ | 164.262±8.035^b^ | 167.337±8.343^b^ | 133.778±4.987^c^ | 248.935±15.513^b^ |
| D8 | 2-Nonanone | 1390 | 64.742±4.635^c^ | 99.684±8.129^a^ | 105.246±8.134^bc^ | 119.545±9.564^a^ | 94.792±7.64^c^ | 170.939±14.703^b^ |
| D9 | 3-hydroxy-2-Butanone | - | 79.27±6.088^d^ | 100.092±7.618^c^ | 98.318±7.993^b^ | 158.297±13.439^b^ | 77.882±5.949^d^ | 240.047±20.624^c^ |
| E1 | ACETIC ACID, METHYL ESTER | 828 | 0 | 0 | 0 | 0 | 0 | 0 |
| E2 | Pentanoic acid, ethyl ester | 1134 | 0 | 0 | 0 | 0 | 1.856±0.128^b^ | 1.703±0.112^b^ |
| E3 | Butanoic acid, pentyl ester | 1305 | 0 | 9.892±0.931^b^ | 11.118±0.129^b^ | 12.799±0.297^c^ | 13.289±0.346^d^ | 0 |
| E4 | acetic acid, 2- methyl-propyl ester | 1012 | 50.895±3.251^a^ | 41.289±3.146^a^ | 28.706±1.888^c^ | 0 | 0 | 0 |
| E5 | 1,2-Benzenedicarboxylic acid-bis(2-methylpropyl) ester | 2536 | 8.845±0.827^c^ | 9.278±0.87^d^ | 11.499±0.167^b^ | 0 | 0 | 0 |
| E6 | Acetic acid, propyl ester | 973 | 0 | 0 | 37.657±2.783^b^ | 44.073±3.424^b^ | 55.489±3.71^b^ | 64.513±4.612^b^ |
| E7 | Butyrolactone | 1632 | 10.464±0.663^a^ | 11.215±0.739^b^ | 13.306±0.948^a^ | 15.352±1.152^a^ | 23.014±1.918^a^ | 30.103±2.627^a^ |
| E8 | 9-Decenoic acid, ethyl ester | 1694 | 55.763±4.337^b^ | 113.734±9.282^b^ | 114.583±9.367^b^ | 136.08±11.517^b^ | 0 | 0 |
| E9 | Nonanoic acid, ethyl ester | 1531 | 23.844±2.001^b^ | 48.871±4.504^a^ | 46.656±4.283^b^ | 60.599±4.821^a^ | 57.733±4.534^c^ | 0 |
| E10 | 2-hydroxy-Propanoic acid ethyl ester | 1347 | 0 | 115.76±9.485^a^ | 158.451±13.754^a^ | 130.54±10.963^a^ | 261.165±22.736^b^ | 529.157±44.545^c^ |
| E11 | Butanoic acid, ethyl ester | 1035 | 679.421±59.571^a^ | 847.309±76.36^a^ | 1053.065±89.915^a^ | 1126.374±97.245^a^ | 1522.19±134.84^a^ | 1886.925±169.412^a^ |
| E12 | Heptanoic acid, ethyl ester | 1331 | 25.112±2.128^b^ | 44.509±4.068^a^ | 47.48±4.365^b^ | 51.756±3.937^b^ | 60.83±4.844^b^ | 83.184±7.079^c^ |
| E13 | Decanoic acid, ethyl ester | 1638 | 201.971±16.816^ab^ | 345.799±30.299^b^ | 320.687±27.788^b^ | 448.774±38.895^ab^ | 403.16±34.334^c^ | 667.819±58.411^a^ |
| E14 | Hexanoic acid, ethyl ester | 1233 | 1235.082±108.116^a^ | 1386.58±123.266^a^ | 1568.558±137.575^a^ | 1665.126±147.232^ab^ | 2218.631±196.182^b^ | 3958.999±370.219^a^ |
| E15 | 1-Butanol, 3-methyl-, acetate | 1122 | 656.394±57.268^a^ | 489.67±42.985^a^ | 537.809±45.41^a^ | 658.017±57.431^b^ | 677.098±59.339^b^ | 862.905±77.92^bc^ |
| E16 | Acetic acid, butyl ester | 1074 | 140.598±11.969^b^ | 143.098±12.219^b^ | 81.431±6.904^d^ | 67.999±5.561^d^ | 54.66±4.227^d^ | 49.439±4.561^d^ |
| E17 | Acetic acid, 2-phenylethyl ester | 1813 | 60.092±4.77^b^ | 96.131±8.374^b^ | 98.221±8.583^b^ | 99.34±8.695^b^ | 72.922±6.053^c^ | 64.26±5.187^d^ |
| E18 | Octanoic acid, ethyl ester | 1435 | 160.911±14^a^ | 196.741±17.583^a^ | 198.878±17.797^a^ | 174.951±15.404^ab^ | 161.367±14.046^b^ | 158.016±13.711^b^ |
| E19 | Acetic acid ethyl ester | 888 | 392.895±35.009^d^ | 440.441±38.062^d^ | 465.04±40.522^d^ | 485.991±42.617^c^ | 351.388±30.858^c^ | 331.268±29.394^d^ |
| F1 | Benzene ethenyl | 1261 | 31.839±2.801^b^ | 57.894±4.55^b^ | 56.976±4.459^a^ | 55.87±4.348^a^ | 51.474±3.908^b^ | 94.809±8.242^a^ |

|  |  |  | CSM:CSS 2:1 | | | | | |
| --- | --- | --- | --- | --- | --- | --- | --- | --- |
|  | compound | RI | CSM:CSS 2:1-0 | CSM:CSS 2:1-10 | CSM:CSS 2:1-20 | CSM:CSS 2:1-30 | CSM:CSS 2:1-40 | CSM:CSS 2:1-50 |
| A1 | 2-Furanmethanol | 1660 | 0 | 0 | 0 | 0 | 0 | 0 |
| A2 | 1-nonanol | 1660 | 0 | 0 | 0 | 0 | 0 | 0 |
| A3 | 1-Octanol | 1557 | 0 | 0 | 5.191±0.521^a^ | 0 | 0 | 0 |
| A4 | 2,3-Butanediol | 1543 | 0 | 0 | 0 | 0 | 1.591±0.198^b^ | 3.781±0.417^b^ |
| A5 | 3-methyl-1-Pentanol | - | 5.97±0.582^a^ | 6.662±0.651^b^ | 6.652±0.688^ab^ | 6.657±0.689^a^ | 6.678±0.691^ab^ | 0 |
| A6 | 1-Pentanol | 1250 | 0 | 0 | 0 | 0 | 1.536±0.161^c^ | 6.261±0.673^c^ |
| A7 | 2-Pentanol | 1119 | 0 | 0 | 2.965±0.269^d^ | 1.914±0.163^d^ | 0 | 0 |
| A8 | 1-Hexanol | 1355 | 0 | 0 | 0 | 2.181±0.239^b^ | 29.852±2.715^b^ | 41.703±3.9^b^ |
| A9 | 3-(methylthio)-1-Propanol | 1719 | 0 | 0 | 0 | 0 | 9.364±0.899^b^ | 11.096±1.373^b^ |
| A10 | 1-Butanol | 1142 | 1.349±0.171^c^ | 2.522±0.288^a^ | 2.789±0.315^a^ | 3.148±0.351^b^ | 0 | 0 |
| A11 | 2-Nonanol | 1521 | 19.331±1.965^a^ | 15.401±1.572^b^ | 13.092±1.341^b^ | 12.083±1.24^b^ | 10.873±1.119^c^ | 8.967±0.929^c^ |
| A12 | 1,2-Propanediol | 1600 | 5.677±0.595^a^ | 4.044±0.431^b^ | 4.56±0.483^c^ | 4.683±0.495^c^ | 5.378±0.565^d^ | 75.677±6.541^c^ |
| A13 | 2-methyl-1-Propanol | - | 109.22±7.949^b^ | 92.812±6.308^c^ | 131.34±10.161^b^ | 113.528±8.38^b^ | 156.378±12.665^b^ | 212.093±18.236^b^ |
| A14 | 2-Heptanol | 1320 | 19.767±2.008^a^ | 23.786±2.41^a^ | 24.243±2.455^b^ | 28.378±2.869^a^ | 33.742±3.405^a^ | 68.109±6.842^a^ |
| A15 | 3-methyl- 2-Butanol | - | 7.827±0.74^d^ | 5.243±0.481^b^ | 16.584±1.615^a^ | 7.435±0.701^c^ | 6.956±0.653^a^ | 5.241±0.481^b^ |
| A16 | Benzeneethanol | 1906 | 316.677±29.282^a^ | 285.762±26.19^b^ | 316.632±29.277^ab^ | 280.854±25.699^bc^ | 499.114±47.525^a^ | 496.293±47.243^a^ |
| A17 | ISO AMYL ALCOHOL | 1209 | 1759.912±132.009^a^ | 1777.02±133.72^a^ | 1983.701±154.388^a^ | 1578.918±113.91^b^ | 1384.994±94.517^c^ | 1202.764±76.294^b^ |
| B1 | 2-furan-carboxaldehyde | 1461 | 0 | 0 | 0 | 0 | 0 | 0 |
| B2 | 2-Hexenal | 1090 | 0 | 0 | 0 | 0 | 0 | 0 |
| B3 | Octanal | 1289 | 0 | 0 | 0 | 0 | 0 | 10.552±0.987^a^ |
| B4 | Hexanal | 1083 | 51.948±4.522^a^ | 0 | 0 | 0 | 0 | 58.08±5.135^a^ |
| B5 | Nonanal | 1391 | 16.527±1.161^b^ | 14.239±0.932^b^ | 6.388±0.601^c^ | 1.68±0.13^e^ | 20.714±2.033^b^ | 23.652±2.327^b^ |
| B6 | Acetaldehyde | 702 | 1.341±0.115^d^ | 1.278±0.109^b^ | 2.071±0.188^b^ | 3.974±0.378^ab^ | 3.49±0.33^a^ | 2.088±0.19^b^ |
| C1 | 2-ethyl-Butanoic acid | - | 0 | 0 | 0 | 0 | 0 | 0 |
| C2 | 2-oxo-Propanoic acid | - | 3.781±0.401^b^ | 3.271±0.35^c^ | 3.013±0.324^c^ | 2.893±0.312^c^ | 2.782±0.301^c^ | 2.384±0.261^b^ |
| C3 | Pentanoic acid | 1733 | 0 | 0 | 0 | 0 | 0 | 0 |
| C4 | 2-methyl-Butanoic acid | - | 40.892±3.161^ab^ | 47.981±3.87^a^ | 55.284±4.6^a^ | 65.289±5.601^a^ | 74.62±6.534^a^ | 88.901±7.962^ab^ |
| C5 | Propanoic acid | 1535 | 0 | 0 | 0 | 0 | 0 | 0 |
| C6 | Heptanoic acid | 1950 | 0 | 0 | 11.101±0.974^d^ | 15.268±1.391^a^ | 17.966±1.661^a^ | 2.449±0.222^b^ |
| C7 | Nonanoic acid | 2171 | 15.915±1.495^b^ | 10.492±0.952^d^ | 17.266±1.63^d^ | 14.959±1.399^d^ | 0 | 0 |
| C8 | 3-methyl-Butanoic acid | - | 52.049±4.829^a^ | 66.055±6.23^a^ | 48.389±4.463^a^ | 36.702±3.294^a^ | 0 | 0 |
| C9 | Decanoic acid | 1097 | 82.117±6.54^a^ | 79.624±6.29^a^ | 78.323±6.16^a^ | 79.421±6.27^ab^ | 116.855±10.014^a^ | 92.959±7.624^bc^ |
| C10 | Butanoic acid | 1625 | 177.439±14.372^b^ | 181.282±14.756^b^ | 201.709±16.799^b^ | 174.637±14.092^c^ | 133.711±9.999^c^ | 112.051±7.833^d^ |
| C11 | Hexanoic acid | 1846 | 56.843±4.66^c^ | 67.141±5.69^b^ | 710.406±70.017^a^ | 86.299±7.606^b^ | 74.878±6.464^b^ | 69.618±5.938^b^ |
| C12 | Octanoic acid | 2060 | 28.854±2.361^b^ | 31.612±2.637^b^ | 32.524±2.728^b^ | 35.396±3.016^b^ | 36.53±3.129^b^ | 38.505±3.327^b^ |
| C13 | Acetic acid | 1449 | 103.537±9.33^d^ | 141.769±13.153^c^ | 151.111±14.087^c^ | 142.464±13.222^c^ | 127.547±11.731^d^ | 112.392±10.215^d^ |
| C14 | 2-methyl-Propanoic acid | - | 80.54±6.73^a^ | 82.719±6.948^a^ | 85.908±7.267^a^ | 68.112±5.487^ab^ | 75.941±6.27^a^ | 74.299±6.106^b^ |
| D1 | 3-Heptanone | 1161 | 0 | 0 | 0 | 0 | 0 | 0 |
| D2 | dihydro-2-methyl-3(2H)-Furanone | - | 0 | 0 | 0 | 0 | 0 | 0 |
| D3 | 2-Octanone | 1287 | 0.892±0.085^b^ | 0.981±0.094^b^ | 1.098±0.1^b^ | 1.156±0.106^b^ | 1219±110.909^a^ | 1.314±0.121^b^ |
| D4 | 2-Pentanone | 981 | 0 | 6.601±0.571^d^ | 5.082±0.419^d^ | 4.298±0.341^d^ | 3.091±0.22^c^ | 3.012±0.212^b^ |
| D5 | 2-Undecanone | 1598 | 0 | 22.045±1.768^bc^ | 22.949±1.858^b^ | 0 | 0 | 0 |
| D6 | 1-hydroxy-2-Propanone | - | 8.678±0.731^b^ | 6.544±0.517^b^ | 2.021±0.201^d^ | 16.062±1.469^b^ | 24.798±2.343^a^ | 30.382±2.901^bc^ |
| D7 | 2-Heptanone | 1182 | 157.678±11.377^a^ | 157.964±11.405^ab^ | 178.907±9.5^b^ | 145.597±6.169^c^ | 249.178±15.537^b^ | 260.916±16.711^b^ |
| D8 | 2-Nonanone | 1390 | 104.783±8.087^a^ | 106.235±8.233^a^ | 116.562±9.265^ab^ | 115.13±9.122^ab^ | 159.556±13.565^b^ | 165.405±14.15^b^ |
| D9 | 3-hydroxy-2-Butanone | - | 169.223±14.531^b^ | 90.016±7.163^c^ | 33.593±2.376^c^ | 170.709±14.68^b^ | 303.93±26.112^b^ | 352.669±30.986^b^ |
| E1 | ACETIC ACID, METHYL ESTER | 828 | 0 | 0 | 0 | 0 | 0 | 0 |
| E2 | Pentanoic acid, ethyl ester | 1134 | 0 | 0 | 0 | 0 | 1.433±0.085^c^ | 1.245±0.067^cd^ |
| E3 | Butanoic acid, pentyl ester | 1305 | 0 | 6.368±0.579^c^ | 7.798±0.722^c^ | 10.525±0.07^d^ | 32.234±2.24^b^ | 33.438±2.361^b^ |
| E4 | acetic acid, 2- methyl-propyl ester | 1012 | 0 | 0 | 0 | 0 | 32.797±2.297^b^ | 85.467±6.708^a^ |
| E5 | 1,2-Benzenedicarboxylic acid-bis(2-methylpropyl) ester | 2536 | 0 | 0 | 0 | 0 | 0 | 0 |
| E6 | Acetic acid, propyl ester | 973 | 40.296±3.047^b^ | 50.314±3.192^a^ | 40.577±3.075^b^ | 50.509±3.212^b^ | 0 | 0 |
| E7 | Butyrolactone | 1632 | 0 | 0 | 0 | 0 | 19.294±1.546^b^ | 21.635±1.781^b^ |
| E8 | 9-Decenoic acid, ethyl ester | 1694 | 68.401±5.601^b^ | 66.065±5.368^c^ | 93.268±8.088^c^ | 141.06±12.015^b^ | 0 | 0 |
| E9 | Nonanoic acid, ethyl ester | 1531 | 38.294±3.446^a^ | 38.684±3.485^b^ | 44.325±4.05^b^ | 55.221±4.283^a^ | 6.28±0.57^d^ | 0 |
| E10 | 2-hydroxy-Propanoic acid ethyl ester | 1347 | 41.291±3.746^d^ | 53.772±4.138^d^ | 93.207±8.082^c^ | 95.311±8.292^b^ | 668.802±58.509^a^ | 1029.378±87.546^a^ |
| E11 | Butanoic acid, ethyl ester | 1035 | 524.204±44.049^b^ | 672.318±58.861^b^ | 916.436±83.273^b^ | 997.566±91.386^b^ | 1213.699±105.978^b^ | 1461.085±130.717^b^ |
| E12 | Heptanoic acid, ethyl ester | 1331 | 33.34±2.951^a^ | 40.687±3.686^a^ | 57.495±4.511^a^ | 78.045±6.566^a^ | 95.68±8.329^a^ | 122.291±10.138^a^ |
| E13 | Decanoic acid, ethyl ester | 1638 | 182.7±16.179^b^ | 196.608±17.57^c^ | 275.51±24.17^c^ | 411.747±35.193^b^ | 774.757±69.105^a^ | 716.403±63.269^a^ |
| E14 | Hexanoic acid, ethyl ester | 1233 | 798.122±71.441^b^ | 860.794±77.708^b^ | 1120.899±96.698^b^ | 1445.615±129.17^bc^ | 3368.015±311.121^a^ | 3292.345±303.554^b^ |
| E15 | 1-Butanol, 3-methyl-, acetate | 1122 | 437.713±37.789^b^ | 508.837±42.513^a^ | 397.009±35.42^c^ | 381.089±33.828^c^ | 625.974±54.226^b^ | 928.123±84.441^b^ |
| E16 | Acetic acid, butyl ester | 1074 | 109.509±8.86^c^ | 117.383±9.647^c^ | 155.318±13.441^b^ | 123.851±10.294^c^ | 99.281±8.689^b^ | 64.26±5.187^d^ |
| E17 | Acetic acid, 2-phenylethyl ester | 1813 | 110.671±8.976^a^ | 119.913±9.9^a^ | 124.685±10.378^a^ | 115.148±9.424^a^ | 103.759±8.285^b^ | 96.427±8.404^c^ |
| E18 | Octanoic acid, ethyl ester | 1435 | 154.936±13.403^a^ | 160.147±13.924^bc^ | 163.909±14.3^b^ | 147.785±12.688^c^ | 124.775±10.387^c^ | 115.152±9.424^c^ |
| E19 | Acetic acid ethyl ester | 888 | 435.466±37.565^cd^ | 456.872±39.705^d^ | 467.797±40.798^d^ | 492.716±43.29^c^ | 528.042±44.433^b^ | 530.629±48.926^c^ |
| F1 | Benzene ethenyl | 1261 | 66.545±5.416^a^ | 75.543±6.315^a^ | 63.297±5.091^a^ | 48.428±4.46^a^ | 52.699±4.031^b^ | 47.654±4.382^c^ |

Data are expressed as the mean ± standard deviation from three replicate analyses (n = 3) of three replicate samples. a-e indicates that there are significant differences between different strains on the same day between the samples (P < 0.05). CS， CSM，CSS，CSM:CSS 1:1, CSM:CSS 1:2 and CSM:CSS 2:1 represent cheeses fermented by Commercial Stater (Control group), *K. marxianus* B13-5, *S. cerevisiae* DL6-20, *K. marxianus* B13-5: *S. cerevisiae* DL6-20 1:1, *K. marxianus* B13-5: *S. cerevisiae* DL6-20 1: 2, *K. marxianus* B13-5: *S. cerevisiae* DL6-20 2:1.
